# Global Gridded Nitrogen Indicators: Influence of Crop Maps

**DOI:** 10.1029/2020GB006634

**Published:** 2020-12-07

**Authors:** K. Kaltenegger, W. Winiwarter

**Affiliations:** ^1^ International Institute for Applied Systems Analysis (IIASA) Laxenburg Austria; ^2^ Institute of Environmental Engineering University of Zielona Góra Zielona Góra Poland

**Keywords:** nitrogen cycling, biogeochemical cycles, processes and modeling, nutrients and nutrient cycling

## Abstract

Displaying Nitrogen (N) indicators on a global grid poses unique opportunities to quantify environmental impacts from N application in different world regions under a variety of conditions. Such calculations require the use of maps showing the geo‐spatial distribution of crop production. Although there are several crop maps in the scientific literature to choose from, the consequences of this choice for the calculation of N indicators still need to be evaluated. In this study we analyze the differences in results for N Use Efficiency (NUE) and N surplus calculated on the global scale using two different crop maps (SPAM and M3). For our calculations we used publicly available statistical and literature data combined with each crop map and carefully traced the origins of the differences in the results. Our results showed that the regions most affected by discrepancies caused by differences in crop maps (yields and physical area) are Central Asia and the Russian Federation, Australia and Oceania, and North Africa. However, we also found that the inclusion or exclusion of grass crops influences the results, as does the aggregation of crops to categories. Considering all these differences, we note that M3 seems to provide the more plausible results for the calculation of N indicators. Our analysis not only highlights the importance of determining the critical parameters for N indicator calculation, but also allows key parameters connected with N use and overuse to be identified on the global scale.

## Key Points

- Parameters like N surplus and NUE values are highly sensitive to the choice of crop map
- Assumptions on grass crops also strongly influence N surplus and NUE
- Least bias in N indicators has been noted when using the M3 crop map

## 1 Introduction

Nitrogen (N) compounds are essential plant nutrients. They contribute to increased crop yields and are thus essential in terms of meeting the food needs of the human population (Erisman et al., 2018). There are not enough naturally occurring N compounds to fertilize the amount of food crops required to meet world demand. Thus, about half the N compounds used in crop cultivation currently come from the Haber‐Bosch process for the production of mineral fertilizer (Smil, 1999).

The addition of synthetic N compounds to the soil as fertilizers significantly alters the N cycle (Fowler et al., 2013). While increased N input to soils can have local effects such as eutrophication of water bodies or soil acidification, it can also produce regional and global effects due to the formation of nitrous oxide (N_2_O). This greenhouse gas has a global warming potential that is 300 times stronger than that of CO_2_. Emissions of N_2_O from agriculture are on the rise, and the impacts of this increase are most probably underestimated (Intergovernmental Panel on Climate Change [IPCC], 2020; Thompson et al., 2019). The human alteration of the N cycle and the need to limit emissions have been addressed on the global level by Rockström et al. (2009) who developed the concept of the nine “planetary boundaries” for essential global parameters: These define the “safe operating space” for each of these parameters within which the functioning and resilience of the Earth system can be sustained. The concept has been updated to include a revision of the boundaries for biogeochemical processes, which is the parameter containing the N and Phosphorus (P) cycles (De Vries et al., 2013; Steffen et al., 2015). The N cycle is one of only two parameters where the Earth system has already overshot the safe operating space. This calls for action to decrease N input.

Indicators such as N surplus (balance of N inputs and outputs) or N Use Efficiency (NUE: N outputs divided by inputs) have been developed to display major N flows. They are key for the development of policies to manage the globally increased N input (Meisinger et al., 2008; Winiwarter and the Expert Panel on Nitrogen Budgets, 2016). When strategies for N management are being developed, a target NUE or N surplus is set. Historic and current NUE and N surpluses are calculated to monitor the evolution of N management through time. As N surplus has multiple impacts, it is relevant to complement a global limit or target with regional boundaries so that the local impacts of N surplus or deficiency can be considered (de Vries et al., 2013). This can be achieved by calculating N indicators such as N surplus and NUE on different scales—global (Galloway et al., 2004), country (Lassaletta et al., 2014; Leip et al., 2011), or regional (Zhang et al., 2015). Being spatially explicit provides the opportunity to simultaneously observe N flows on several scales through upscaling or downscaling and thus to describe spatial variations of N globally, regionally among countries, and also within countries (Bouwman et al., 2017; Liu et al., 2010; West et al., 2014). The increased abundance of spatially explicit N input data has paved the way for such calculations. These data include atmospheric N deposition as well as gridded data for livestock and crop distribution from which other N inputs like manure N application, crop‐specific Biological Nitrogen Fixation (BNF), crop‐specific mineral fertilizer application, and N output can be determined (Lamarque et al., 2013; Lu & Tian, 2017; Zhang et al., 2017; Xu et al., 2019).

The calculation of N indicators generally comes with quite large uncertainties. Knowledge of these uncertainties is of great value, especially for developing or assessing N management policies. While unconsidered uncertainties may lead to wrong conclusions and ineffective N management, the evaluation of uncertainties provides information on the weakness of an N budget. This enables the adoption of conservative assumptions or safety factors where necessary (Oenema et al., 2003). For country and regional NUE and N surplus calculations, uncertainties mainly originate from differing values for input parameters such as mineral fertilizer, BNF, N deposition, N content of crops, and manure N, as well as different system definitions (Zhang et al., 2020). Moreover, when these indicators are calculated on a spatially explicit basis, there could be additional uncertainties that need to be understood but have not yet been discussed.

One additional uncertainty could originate in the underlying crop maps used. Crop maps, such as the Spatial Production Allocation Model (SPAM) and M3, allocate attributes of crop production (harvested area, physical areas, and production) to geographically explicit areas (grid cells) (Monfreda et al., 2008; You et al., 2014). They are used to spatially distribute crop‐specific country or regional data such as mineral fertilizer and BNF, which account for more than 70% of all N inputs as well as N content, which regulate the amount of N output (Liu et al., 2010; Zhang et al., 2020). They thus play a crucial role in the spatial distribution of N surplus and NUE. However, there are several different crop maps available, showing substantial differences in terms of cropland extent, harvested areas, and production entries with variable, region‐dependent magnitude (Anderson et al., 2015). Knowledge of how these differences affect the calculation of N indicators is an essential foundation for an informed choice of crop map and an adequate interpretation of the indicators. To contribute to this foundation, we calculated global, spatially explicit N surplus and NUE on two different crop maps and closely observed the differences between them and their causes. This paper provides information on the origin and magnitude of the differences we found and the regions most affected by them; it proposes a choice of crop map best suited for our calculation of global gridded N indicators.

## 2 Methods

A plethora of definitions exist for N surplus and NUE, depending on the boundaries set for the system under consideration. For a proper definition of these, the N inputs and N outputs being considered need to be decided upon first (Ladha et al., 2005; Oenema et al., 2003). In this study, the inputs included are mineral fertilizer, manure application (with volatilization losses and losses due to storage being subtracted), BNF, and N deposition. The outputs include the N contained in crop harvest. Mineralization, the decomposition to inorganic material, is excluded. Crop residues are also excluded, as they are assumed to stay within the system. We follow the definition of “soil surface N budgets,” as described by Oenema et al. (2003), and apply it solely to cropland areas to highlight the effect of crop maps. We use only the budget terms (N inputs and outputs) included in this definition for N surplus and NUE calculation.

N surplus (N_BS_) is calculated by subtracting N outputs from N inputs (1). NUE is calculated by dividing the N contained in the crop harvest by all inputs (2). Where possible, data on the components of the N indicator calculations were taken from statistics.

(1) $$N_{\mathrm{BS}}=\frac{\text{Mineral Fertilizer}+\mathrm{BNF}+N\;\text{Deposition}+\text{Manure}\;N-N\;\text{Harvest}}{\text{Cropland Area}}$$

(2) $$\mathrm{NUE}=\frac{N\;\text{Harvest}}{\text{Mineral Fertilizer}+\mathrm{BNF}+N\;\text{Deposition}+\text{Manure}\;N}$$

(3) $$N\;\text{Harvest}=P_{\text{crop},\text{cell},c}\times {\mathrm{Nc}}_{\text{crop}}$$

(4) $$\text{Mineral Fertilizer}={\mathrm{HA}}_{\text{crop},\text{cell},c}\times {\mathrm{rF}}_{\text{crop},\text{cell},c,\mathrm{HA}}$$

(5) $$\mathrm{BNF}={\mathrm{HA}}_{\text{crop},\text{cell},c}\times {\text{rBNF}}_{\text{crop},\text{cell},c,\mathrm{HA}}$$

(6) $$N\;\text{Deposition}={\mathrm{ND}}_{\text{cell}}\times {\text{frCL}}_{\text{cell}}$$

(7) $$\text{Manure}\;N={\mathrm{MN}}_{\text{cell}}\times {\text{frCL}}_{\text{cell}}$$

P_crop, cell, c_: … Crop‐ and country‐specific (c) crop production (kg)
Nc_crop_: … Crop‐specific N content (1)
HA_crop, cell, c_: … Crop‐ and country‐specific harvested area (ha)
rF_crop, cell, c, HA_: … Crop‐ and country‐specific mineral fertilizer rate per harvested area (kg/ha)
rBNF_crop, cell, c, HA_: … Crop‐ and country‐specific BNF rate per harvested area (kg/ha)
ND_cell_: … N Deposition in grid cell (kg)
frCL_cell_: … Fraction of cropland of total cell land area (1)
NM_cell_: … Manure N in grid cell (kg)

### 2.1 Crop Maps

To spatially distribute these statistical data, two crop maps—M3 (Monfreda et al., 2008) and SPAM (You et al., 2014)—were employed. SPAM uses several different inputs (spatial and non‐spatial) to determine the spatial distribution of harvested area and yield for 42 different crop types. Crop production statistics are taken from Agro‐MAPS (Food and Agricultural Organization of the United Nations et al., 2006) and are complemented with sub‐national statistics collected from local sources. The statistical data are then combined with a classified land cover image derived from the combination of different land cover data sets and information on crop‐specific suitability based on landscape data, climate, and soil conditions, together with crop prices and population data, to create a map of estimated crop‐specific area shares per pixel. This map is then combined with another map of area shares from a prior estimate or another crop map using a cross‐entropy approach. Using this approach, for each pixel, the area share where the cross entropy—representing the discrepancy between the estimated share and prior share map—is minimal, is selected to create the final product (You & Wood, 2006).

The M3 crop map developed by Monfreda et al. (2008) is based on data that are similar to those of the SPAM crop map, and it also uses Agro‐MAPS supplemented with sub‐national statistical data on harvested area and yields of 175 different crop types. These data are then distributed on a cropland map according to each crop's share of total cropland per political unit. Harvested areas were adjusted using multiple cropping potentials derived from the Global Agro‐Ecological Zones Agroecological Zone (GAEZ) assessments developed by the Food and Agricultural Organization of the United Nations (FAO) and the International Institute for Applied Systems Analysis (IIASA) (IIASA/FAO, 2012). The cropland map was taken from Ramankutty et al. (2008) who used a combination of two land cover products to spatially disaggregate statistical data on cropland. Cropland is defined according to the FAO classification as arable land and permanent crops, including fallow lands and temporary cropland as well as temporary meadows and pastures (FAO, n.d.). Temporary meadows and pastures were assumed to be represented by M3 crops such as alfalfa, grassnes (grass not elsewhere specified), fornes (forage not elsewhere specified), clover, mixed grass, and vetches, hereafter referred to as grass crops.

The procedure of allocating crop production and harvested area in M3 differs substantially from the cross‐entropy approach taken by SPAM. While in M3, crop allocation mainly depends on the cropland area map provided by Ramankutty et al. (2008) and the GAEZ multiple cropping index, SPAM also considers crop prices and population. This can explain differences in crop allocation as well as the use of different statistical sub‐regional data.

As the more recent crop map, SPAM, contained data from the year 2010 and because all other N input data were also available for the year 2010, we chose 2010 as our base year for the comparison. A resolution of 0.5° was chosen. As the M3 crop map uses the year 2000 as base year, data on harvested areas, production, and cropland area were updated to fit country data from the Statistics Division of FAO (FAOSTAT) to make it comparable to the SPAM crop map (FAO, 2019b). For this update, each crop in each cell was multiplied by an update factor, derived from the ratio of FAOSTAT country data, to the aggregated M3 country data (8). The same procedure was used for updating production and harvested areas. As no crop production information for forage or grass crops was found in the FAOSTAT database, the original M3 data was used. Cells with cropland area below 5% were excluded to avoid including outliers.

(8) $${\mathrm{HA}}_{\text{crop},\text{cell}}=\sum_{c}\frac{{\mathrm{HA}}_{\text{crop}\left(\mathrm{FAO}\right),c}}{{\mathrm{HA}}_{\text{crop}\left(M3\right),c}}\times p_{c}\times {\mathrm{HA}}_{\text{crop},\text{cell}}$$

HA_crop, cell_: … Updated cell entry for harvested area for one crop (ha)
HA_crop(FAO), c_: … Country (c) total for one crop in 2010 according to FAO (ha)
HA_crop(M3), c_: … Country (c) total for one crop in 2000 using M3 (ha)
HA_crop, cell, c_: … Cell entry for one crop in 2000 using M3 for the country (c) the cell belongs to (ha)
p_c_: … Share of total land area of cell belonging to country (c) (1)

### 2.2 Mineral Fertilizer

Mineral fertilizer application per crop type (14) and country or region (28) was taken from a report for the year 2010, provided by the International Fertilizer Agency (IFA) (Heffer, 2013; Heffer et al., 2017). Information on fertilizer use per grass crops was only added in the year 2014 when the category “Other Crops” was divided into “Residuals” and “Grass Crops.” As this information was needed for the M3 calculation and therefore the year 2010, a multiplication factor describing the share of “Grass Crops” in “Other Crops” was derived from the 2014 data. This factor was then used on the 2010 crop category “Other Crops” to obtain the share of mineral fertilizer application to grass crops. To distribute mineral fertilizer application spatially, fertilizer use per harvested area, crop type, and country was calculated by first dividing the IFA mineral fertilizer data by the sum of harvested area per crop type and country, aggregated from each crop map. The result was then multiplied by the harvested area per crop type and country in each cell. This procedure could lead to biased mineral fertilizer application because its distribution within a country category is according to shares in harvested area and does not consider country‐specific application rates influenced by agronomic conditions. Due to this bias, mineral fertilizer input was adjusted to fit FAOSTAT annual country totals (FAO, 2019a). FAOSTAT states that mineral fertilizer application includes fertilizer use for crops, livestock (e.g., pasture for grazing), and aquacultures. As land used for aquacultures has only a small share in total land (less than 0.01%) compared to land used for agriculture (more than 35%), fertilizer application in this agricultural practice was considered negligible (FAO, 2019c). However, fertilizer application to pastureland was considered and subtracted using data provided by Lassaletta et al. (2014), providing shares of mineral fertilizer applied to pastureland. NH_3_ and N_2_O volatilization were considered using data on urea share in synthetic fertilizers per country as well as country‐specific NH_3_ volatilization rates for the urea and non‐urea share from the Greenhouse Gas‐Air Pollution Interactions and Synergies (GAINS) model (Amann et al., 2011). N_2_O volatilization rates were also taken from GAINS, with differentiation being made between paddy rice and other crops.

### 2.3 BNF

To distribute BNF, factors of BNF per crop type (for all crops available in a crop map) and country (country differentiation was only available for soybeans) were taken from Herridge et al. (2008) and multiplied with harvested areas from each crop map. For grass crops, average BNF rates were taken from Smil (1999) who differentiates between different forages and grasses, which matches our grass crop definition.

### 2.4 Nitrogen Deposition

To include N deposition, data from the Chemistry‐Climate Model Initiative (CCMI) provided by Tian et al. (2018) were used. As these data show global N deposition (including deposition on other areas such as water bodies, grassland, and infrastructure areas), they are filtered to include only the cropland area given by the respective crop map.

### 2.5 Manure Nitrogen

Spatially explicit data on livestock numbers per livestock system are taken from the Gridded Livestock of the World (Robinson et al., 2014) and combined with N excretion rates per animal type and country from GAINS. Fractions of manure managed and lost during storage per livestock system are taken from Herrero et al. (2013) to calculate manure N application. Country‐specific application rates to cropland were taken from Liu et al. (2010) to calculate the fraction of manure N being applied to cropland only. These were then multiplied by the manure N applied and the cropland area in each grid cell.

### 2.6 Nitrogen Harvest

N content per crop was taken from several sources with most of the data coming from a document provided by the European Expert Panel on Nitrogen Budgets (Winiwarter and the Expert Panel on Nitrogen Budgets, 2016) and data provided by Lassaletta et al. (2014).

### 2.7 Cropland Area

For calculating N surplus at the level of soil surface, the balance (output subtracted from input) is divided by the cropland area it relates to. As M3 only provides information on harvested areas, which include multi‐cropped areas multiple times, physical cropland area for the M3 calculation is taken from Ramankutty et al. (2008) consistent with the approach taken for the development of the M3 crop map. SPAM provides physical cropland area per crop or crop type in addition to harvested areas and production.

Further details concerning the methodology are presented in the supporting information (Texts S1–S6 and Data Set S1).

**Figure 1:**
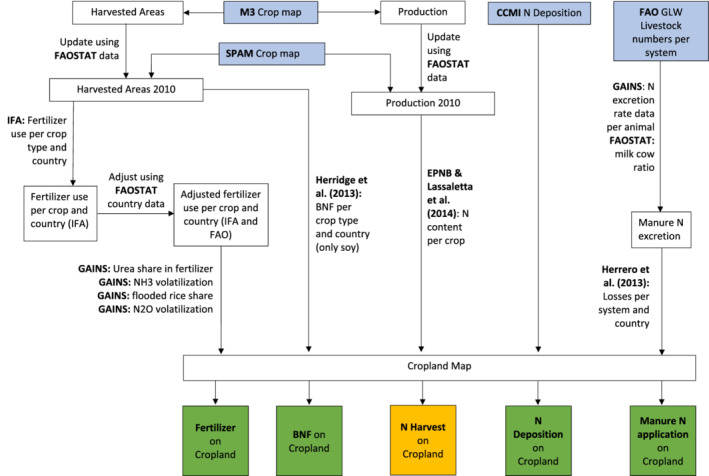
Processing of data to spatially distribute statistical data for the development of four different input maps (Fertilizer, BNF, N deposition, and manure N) and one output map (N harvest) for each crop map. Blue boxes contain mapped input data, green boxes contain N input maps, and the orange box contains the N output map. The cropland maps are either taken from SPAM directly or from Ramankutty et al. (2008) to obtain M3 results.

A summary of data used, their interaction, and the processing undertaken to arrive at the final N input or output is visualized in Figure 1.

## 3 Results

To identify the effects that crop maps and their inherent characteristics have on the NUE calculations at several scales and also to detect differences between the scales, we chose a systematic approach for our analysis. We concentrated first on differences on the global grid, then on the regional level, and finally on the country level.

### 3.1 Global Grid

Our focus, when looking at the global grid, was not only to gain an impression of the global total difference in cropland extent but also to obtain the bigger picture of how innate characteristics of crop maps, such as yield, translate to the calculation of NUE, which of course depends on the yields displayed by the respective crop map.

#### 3.1.1 Cropland Area

We compared cropland area in M3 to cropland area in SPAM and discovered a high discrepancy in cropland extent between the maps, amounting to a difference of 3.6 × 10^6^ km^2^ of cropland area; this can largely be explained by SPAM having no representation of grass and leguminous pasture crops (clover, alfalfa, mixed grass, vetches, and other grasses).

**Figure 2:**
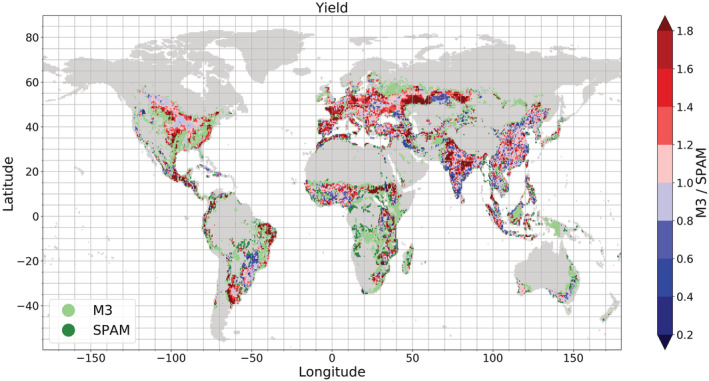
Ratio between M3 and SPAM yield (M3—no grass crops included for comparison) for each 0.5° × 0.5° grid cell. Light green areas indicate areas where only M3 cropland area can be found, and dark green areas indicate areas where only SPAM cropland area can be found.

However, as well as cropland extent there is also a difference in cropland allocation. There are some areas that only one of the crop maps allocates cropland to (see light green or dark green areas in Figure 2). In North America, Ireland, and Northwestern Russia, many areas can be found, where cells only show M3 cropland, whereas in Central Africa, Indonesia, Madagascar, and Papua New Guinea, M3 and SPAM allocate cropland to completely different areas. Altogether, cells only containing M3 cropland make up only 25% of all non‐empty cells, whereas cells only containing SPAM cropland constitute only 8% of all non‐empty cells.

#### 3.1.2 Yield

We compared harvested areas, crop production, and yields (as tons of product per harvested area) for each crop type, deciding to exclude information on harvested area and production of grass crops from M3 to enable a comparison. This was because SPAM, as mentioned above, does not contain information on these crops. Excluding grass crops leads to a good global agreement of crop production (8.2 Gt in M3, 7.8 Gt in SPAM), harvested area (1,322.2 Mha in M3, 1,300.9 Mha in SPAM) and subsequently yield (6.2 t/ha in M3, 6.0 t/ha in SPAM). However, when we took a closer look at the global distribution of yield as the combination of harvested area and production, we saw that different patterns with a regionally changing level of discrepancy between M3 and SPAM remained (Figure 2). In general, there are more grid cells with higher yields in M3 (red cells in Figure 2) than cells with higher yield in SPAM (blue cells in Figure 2) originating from higher crop production in M3 in most regions. This could be explained by higher yields of forage crops, which, in the SPAM data, are only partially included in different crop categories (except for silage maize) or by a different mix of crops within the crop categories.

Within India, SPAM and M3 seem to be allocating different crops to different regions. Although the country sums for crop production and harvested area are very similar in SPAM and M3, the map displays that relatively higher yields to the north of the country are allocated by M3, and to the south by SPAM.

$$\text{Yield}=\frac{\text{Production}}{\text{Harvested}\;\text{Area}}$$

#### 3.1.3 NUE

**Figure 3:**
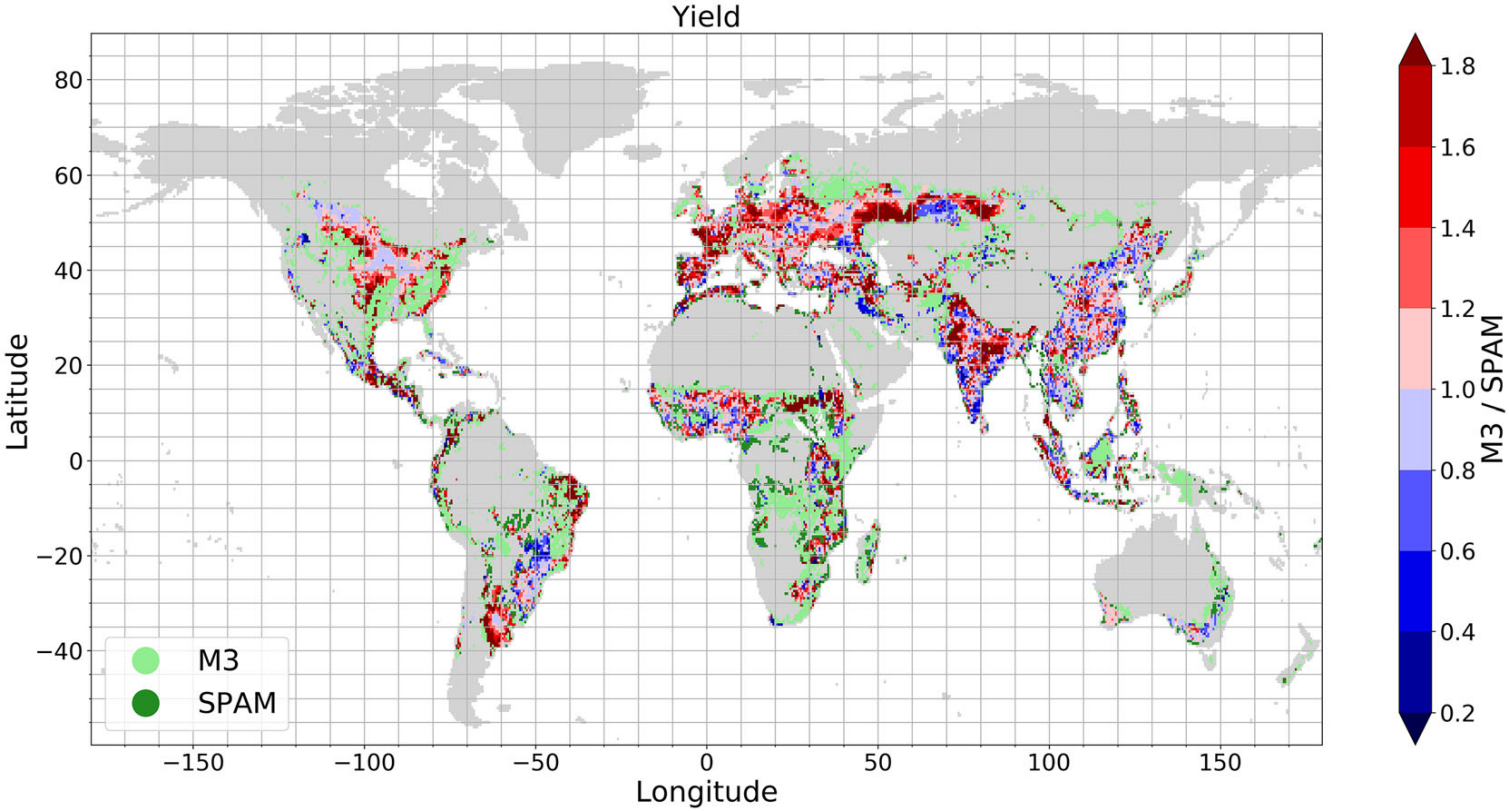
Ratio between nitrogen use efficiency (NUE) calculated with SPAM and M3 crop map (IFA mineral fertilizer and no grass crops in M3 included for comparison). Light green areas indicate areas where only M3 cropland area can be found, and dark green areas indicate areas where only SPAM cropland area can be found.

We quantified NUE according to Equation 2 for each grid cell. Figure 3 displays the ratio between M3‐ and SPAM‐based NUE to identify the differences. The pattern of the ratio of NUE (N harvest/N input) is similar to the pattern of yield ratios (production/harvested area) and is an example of how discrepancies in the original crop‐specific data translate to data calculated from them. The close link between yield ratios and NUE ratios can be explained by both terms being strongly influenced by the respective production amounts and harvested areas (see section 2, Equations 2 and 3). This link between yield and NUE is visible when Figure 2 is compared to Figure 3.

While the patterns on these maps are similar (grid cells with higher SPAM/M3 yield ratios also show higher SPAM/M3‐based NUE ratios), the discrepancy between NUEs is lower, and in certain cases SPAM‐based NUEs may become even higher than when NUEs are derived from M3 compared to the yield ratios. This can be explained by manure N and N deposition entries often being higher for M3, as the M3 cropland area is larger and covers more grid cells. Note that for grid cells that contain animals, which produce manure, but have a cropland area smaller than 5%, manure is ignored as N input. This difference in cropland area is largely driven by grass crops. It was not possible to exclude cropland covered by grass crops because no crop‐specific information was given in physical areas per grid cell for M3. As manure N and N deposition are allocated to a physical area, a separate evaluation of non‐grass crops is not possible. Hence, manure N and N deposition applied to grass crops are also included for M3‐based NUE calculation. This can be seen most clearly in the extensive grass crop areas located in northwestern North America and Western Russia. In these areas or grid cells, where M3 shows higher yields, SPAM‐based calculations show higher NUEs because the M3‐based NUEs are lowered due to higher N inputs.

Additionally, M3 N harvest is often lower compared to SPAM even though M3 production might be higher. This can be explained by M3 often including crops that are not listed individually in SPAM crop categories. A closer look at the crop distribution and N harvest interplay in Southern Spain illustrates this phenomenon. SPAM shows clearly higher results for NUE in this area. As N inputs are very similar for both crop maps, the difference can be attributed to one important crop for the region: olives. While olives are included as single crop in M3, they are included in the crop category “Other Oilseeds” in SPAM. This categorization influences the overall NUE and N surplus calculation, as olives are assigned an N content of 0.2% while “Other Oilseeds” are collectively assigned an N content of 3.9%. As olives make up 89% of total crop production of “Other Oilseeds,” misallocation of N in harvest based on the less detailed specification in SPAM is thus the reason for the observed discrepancy in NUE.

### 3.2 Regional Results

To investigate regional differences more closely, and also the possible reasons for those differences, SPAM‐based NUE and variations of M3‐based NUE and N surplus (grass crops included or excluded) were aggregated to 17 different world regions and results were compared at that level. We discovered that grass crops influence the results on a global level. We thus also took a closer look at their role in regional differences. The NUE comparison allows differences in harvested areas and production between the crop maps to be observed more closely, while for N surplus additional information on cropland area is always included. For both crop maps, the unadjusted version using IFA fertilizer was used to minimize the influence of possible artifacts from additional calculations.

#### 3.2.1 NUE

**Figure 4:**
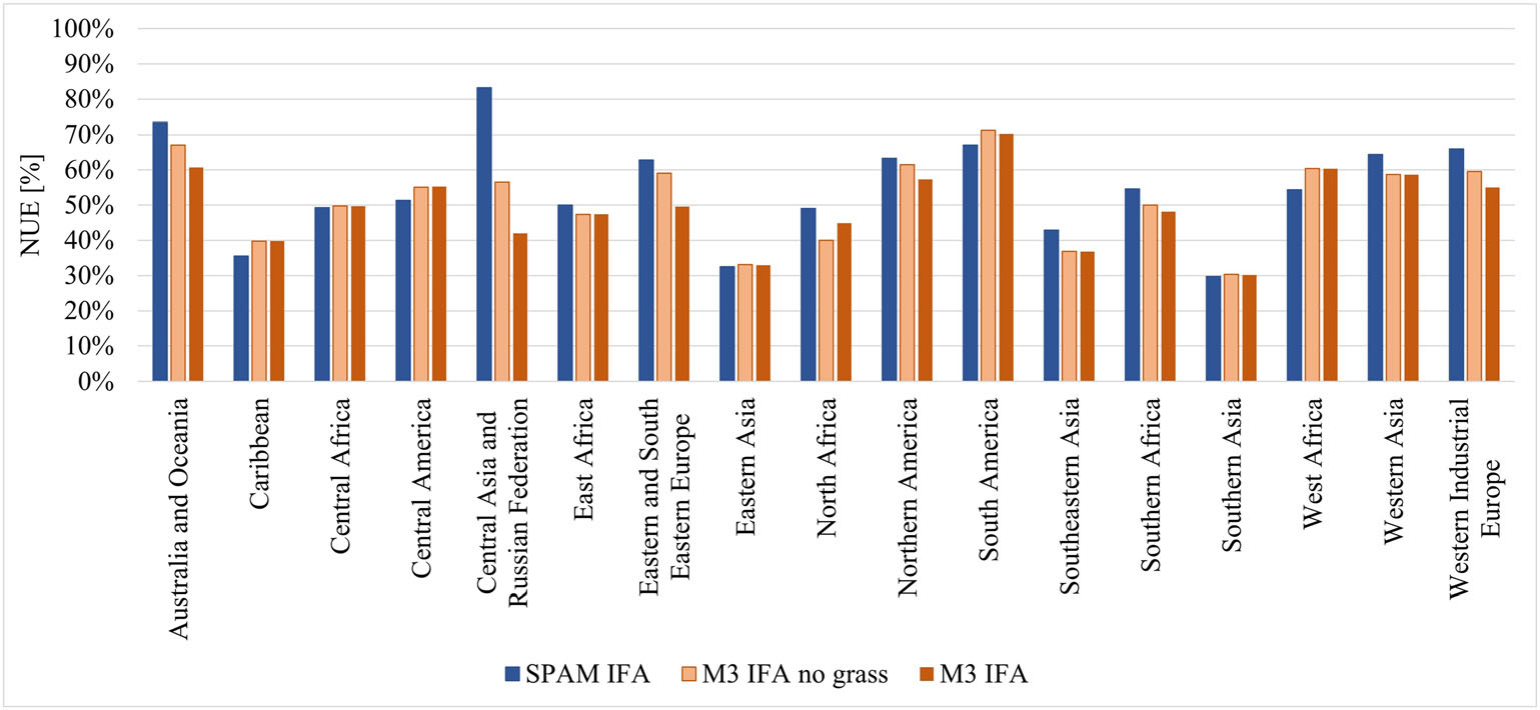
SPAM—and variations of M3‐based NUE calculations for 17 world regions. SPAM IFA—calculations based on SPAM crop map and IFA mineral fertilizer data; M3 IFA no grass—calculations based on M3 crop map, excluding grass crops and IFA mineral fertilizer data, M3 IFA—calculations based on M3 crop map including grass crops and IFA mineral fertilizer data.

We compared SPAM‐based NUE with M3‐based NUE without grass crops but with IFA mineral fertilizer (blue and red bars in Figure 4) to observe crop map‐inherent differences. In general, M3‐based NUE is lower than, or equal to, SPAM‐based NUE. Lower M3‐based NUE can be explained by manure N and N deposition entries on grass crop areas being included in the M3 calculations due to their distribution on cropland area where no crop‐specific differentiation is possible, and not being included in the SPAM‐based calculations. Cropland allocation in the respective map also influences the results, as gridded manure N and N deposition calculated from other sources is filtered to fit the respective cropland distribution.

In Central America, South America, Southern Asia, Western Africa, and the Caribbean, too, more N is harvested in the M3‐based calculations, and the additional N input remains below the additional output. It was noted that increased M3 output has different causes in different regions and different crop categories. In Eastern Asia, Southern Asia, and Western Africa, “Residuals” highly influence the N output discrepancy between the M3‐ and SPAM‐based calculation, as M3 includes high‐yielding forage crops in the “Residuals” category that are not included in SPAM. In Central and South America, M3 assigns more harvested area and a higher yield to soybeans than SPAM does, while in the Caribbean a three times higher fruit yield of 33 t/ha leads to a higher N output in the M3‐based than in the SPAM‐based calculations. In Southern Africa, a production difference in sunflower seeds leads to higher N harvests in SPAM, resulting in a higher SPAM‐based NUE (see Data Set S2 in the supporting information for more details). Despite these differences, SPAM‐ and M3‐based NUE without grass crops are usually within 10% of each other, as can be seen in Figure 4.

As North Africa, Central Asia and the Russian Federation, and Australia and Oceania show larger discrepancies, they were investigated more closely. The explanation for the discrepancies in North Africa is similar to that for the discrepancies found in the south of Spain. As olives with a rather low N content dominate the category “Other Oilseeds,” harvested N in M3 is significantly lower than in SPAM. As M3 allocates more cropland area to North Africa than SPAM does, manure N and N deposition input are higher in M3, adding to the decrease in M3‐based NUE. In Central Asia and the Russian Federation, the discrepancies between SPAM‐ and M3‐based NUE are mainly driven by 1.3 times higher “Wheat” and a 1.7 times higher “Other Cereal” production in SPAM, leading to a 1.3 and 1.9 times higher value of harvested N, respectively, in the SPAM‐based calculation. However, Central Asia and the Russian Federation is also the region with the highest share of grass crops in harvested area, which leads to increased N input from manure N and N deposition, as grass cropland areas cannot be excluded from the calculation. The combination of these effects explains a lower NUE when M3 is used. In Australia and Oceania, higher yield for “Wheat” and “Other Cereal” in SPAM leads to a higher SPAM‐based NUE.

Olives also play a role in Western Industrial Europe, lowering the M3‐based N harvest. A rather high discrepancy in cropland area leads to a higher N deposition input, and a higher production of “Residuals” results in higher BNF values in the M3‐based calculation. All these effects lead to a lower M3‐based NUE. However, as total N input and N output are rather high, the difference between the two NUEs remains low.

#### 3.2.2 N Surplus at Soil Surface Level

**Figure 5:**
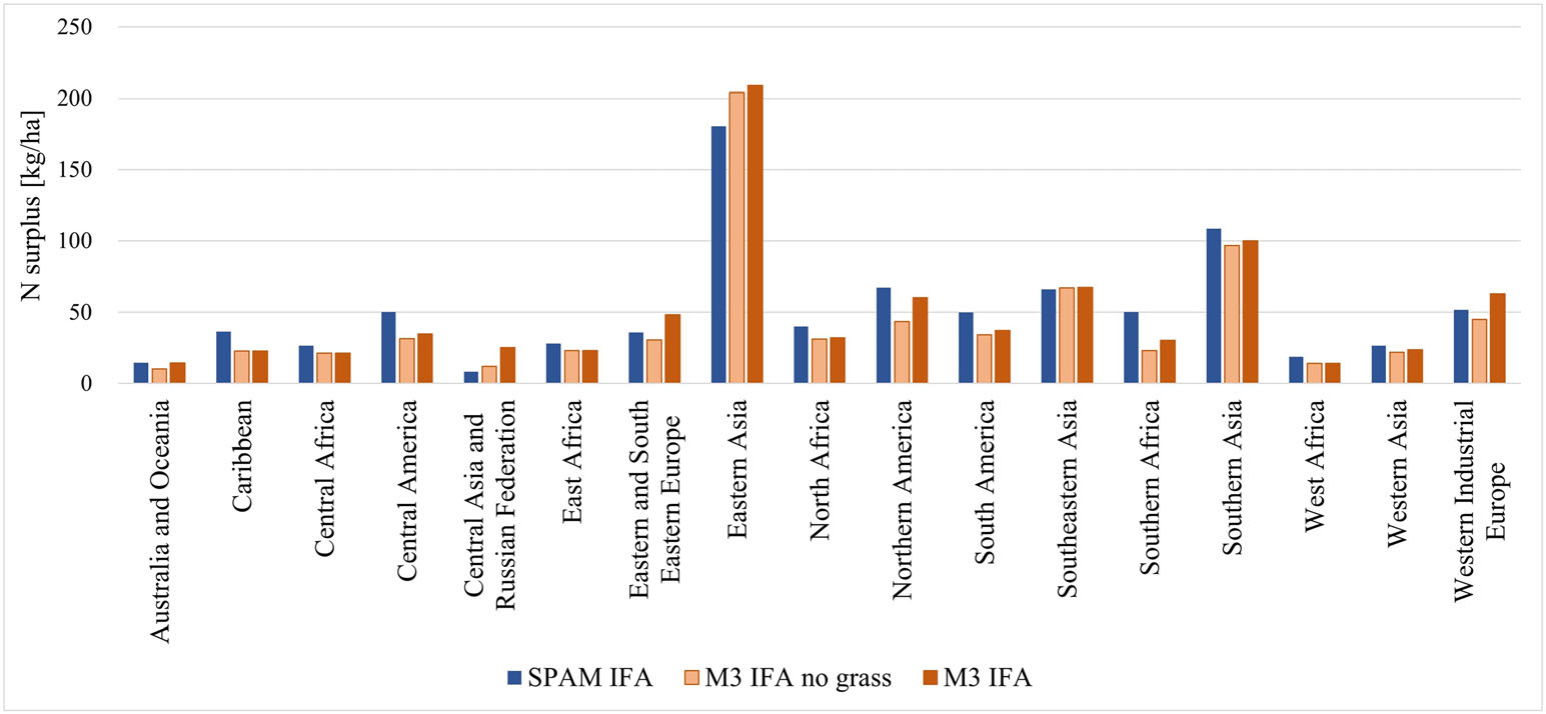
SPAM and variations of M3‐based N surplus calculations for 17 world regions. SPAM IFA—Calculations based on SPAM crop map and IFA mineral fertilizer data; M3 IFA no grass—Calculations based on M3 crop map excluding grass crops and IFA mineral fertilizer data, M3 IFA—Calculations based on M3 crop map including grass crops and IFA mineral fertilizer data.

Figure 5 shows N surplus presented in the same way as NUE. Regions where a low NUE was observed previously, typically show high N surplus (Eastern Asia, Southern Asia), whereas regions with high NUE indicate little N surplus (Australia and Oceania, Central Asia and Russian Federation). When N surplus is compared to NUEs, the influence of cropland area is apparent. In most regions, it decreases the M3‐based results compared to the SPAM‐based results. This is especially true for regions with high discrepancies in cropland area such as Central America, North America, and Southern Africa (Table 1). Eastern Asia is the only region to which SPAM allocates more cropland than M3 does, which leads to lower N excess per hectare when using SPAM compared to all M3‐based variations. In Central Asia and the Russian Federation, the higher SPAM yields for “Other Cereals” and “Wheat,” mentioned above, lead to less N surplus per hectare due to higher N outputs.

**Table 1:** Ratios Between M3 and SPAM of Different Influences on NUE and N Surplus Results and Shares of Grass Crops in Harvested Area

Region	SPAM/M3 cropland ratio	SPAM/M3 manure N ratio	SPAM/M3 N deposition ratio	SPAM/M3 harvested area ratio	SPAM/M3 IFA fertilizer ratio	SPAM/M3 BNF ratio	SPAM/M3 production ratio	SPAM/M3 N harvest ratio	% grass crops in total harvested area
Australia and Oceania	0.92	0.92	0.95	1	1.01	0.94	0.99	1.09	0.05
Caribbean	0.67	1.02	0.97	0.98	0.95	0.99	0.73	0.87	0
Central Africa	0.79	1.07	0.79	0.93	0.92	0.96	0.97	0.92	0
Central America	0.71	0.85	0.71	0.95	1.01	0.94	0.94	0.88	0.09
Central Asia and Russian Federation	0.49	0.82	0.46	1.06	1	0.95	0.99	1.27	0.28
East Africa	0.77	1.01	0.71	1.04	1.02	0.92	1.06	1.01	0
Eastern and South Eastern Europe	0.76	0.98	0.78	0.93	1.03	0.8	0.79	1.03	0.15
Eastern Asia	1.14	0.99	1.23	1	1	0.95	0.99	0.99	0.02
North Africa	0.77	0.87	0.8	0.96	0.99	0.83	0.97	1.15	0.06
Northern America	0.62	0.7	0.65	0.97	1	0.98	0.88	0.97	0.2
South America	0.9	0.94	0.98	1	1	1	0.97	0.93	0.04
Southeastern Asia	0.9	1	0.94	0.99	1	0.99	1.13	1.16	0
Southern Africa	0.43	0.75	0.43	1.01	1	1.04	1.05	1	0.19
Southern Asia	0.92	0.98	1	0.99	1	1	1.01	0.98	0.04
West Africa	0.87	0.96	0.9	0.97	1.01	1	0.95	0.89	0
Western Asia	0.7	0.93	0.77	0.97	0.99	0.93	0.97	1.05	0.05
Western Industrial Europe	0.69	0.91	0.78	0.89	0.98	0.7	0.7	1.03	0.13

*Note*. SPAM—calculations based on SPAM crop map and IFA mineral fertilizer data; M3—calculations based on M3 crop map excluding grass crops and IFA mineral fertilizer data.

#### 3.2.3 Influence of Grass Crops

To understand the bias introduced by the inclusion of grass crops, we compared M3‐based calculations using IFA fertilizer with (M3 IFA) and without grass crops (M3 IFA, no grass). As bias might also derive from adjustment procedures to FAOSTAT fertilizer data, we excluded the FAOSTAT‐based dataset from the intercomparison. In comparing NUEs in Figure 4, one can see how the influence of grass crops changes according to the share of grass crops in a region's total harvested area (Table 1). Generally, despite the high yields of grass crops, their inclusion increases N input because the BNF rate we assigned to them is higher than the BNF rate for most non‐leguminous crops, thus leading to lower NUEs. Exceptions are areas where grass crop yields are very high, such as North and East Africa, Central America, and Western Asia. In these regions, additional N output due to higher yields is higher than the additional N input compared with the respective N input or output without grass crops, leading to higher NUEs. In Northern Africa, clover cultivation in Egypt greatly influences the results due to its high yield (40 t/ha), while in Central America and Western Asia, high‐yielding alfalfa in Mexico (60 t/ha) and Turkey (40 t/ha) is the most influential grass crop. Vetches contribute to a higher NUE when grass crops in East Africa are included. With respect to the differences between soil surface N surplus including or excluding grass crops, the pattern of NUE calculations observed is now the opposite, as shown in Figure 5. When grass crops are included (M3 IFA), N surplus increases due to higher N input.

### 3.3 Country Results

To gain a better impression of small‐scale effects that might not be discovered in the regional analysis, we also compared country results for SPAM‐based NUE using IFA fertilizer and M3‐based NUE using IFA fertilizer and excluding grass crops (Table 2). At least one country per region (either the biggest or the one showing the highest discrepancy to other data) was selected for the comparison.

**Table 2:** Country NUEs Based on the Respective Crop Map and Grass and Forage Crop Shares in Total Harvested Area (Grass and Forage Crop Shares Only Hold for M3)

Country (ISO code)	Region	NUE SPAM IFA	NUE M3 IFA no grass	Grass crops (%)	Forage crops (%)
ARG	South America	77%	87%	12%	4%
AUS	Australia and Oceania	71%	64%	5%	0%
BGR	Eastern and South Eastern Europe	49%	57%	7%	2%
BLZ	Central America	49%	52%	0%	0%
BRA	South America	63%	65%	0%	0%
CAN	Northern America	66%	61%	22%	1%
CHN	Eastern Asia	32%	33%	1%	1%
COG	Central Africa	49%	46%	0%	0%
CUB	Caribbean	30%	26%	0%	0%
CYP	Western Asia	23%	11%	60%	33%
DEU	Western Industrial Europe	81%	71%	3%	27%
EGY	North Africa	37%	34%	17%	0%
ESP	Western Industrial Europe	47%	34%	7%	2%
FIN	Western Industrial Europe	63%	49%	31%	1%
FRA	Western Industrial Europe	77%	77%	12%	12%
GBR	Western Industrial Europe	81%	73%	21%	3%
GHA	West Africa	76%	78%	0%	0%
IDN	Southeastern Asia	38%	37%	0%	0%
IND	Southern Asia	29%	30%	4%	4%
IRL	Western Industrial Europe	72%	38%	40%	2%
ITA	Western Industrial Europe	51%	43%	18%	6%
KEN	East Africa	45%	45%	0%	0%
MEX	Central America	39%	41%	4%	2%
MYS	Southeastern Asia	44%	45%	0%	0%
NZL	Australia and Oceania	182%	160%	18%	36%
PRT	Western Industrial Europe	27%	25%	28%	25%
RUS	Central Asia and Russian Federation	91%	56%	33%	7%
SWE	Western Industrial Europe	72%	57%	42%	0%
TUR	Western Asia	56%	54%	2%	0%
USA	Northern America	63%	62%	20%	2%
ZAF	Southern Africa	56%	51%	21%	0%

*Note*. SPAM IFA—calculations based on SPAM crop map and IFA mineral fertilizer data; M3 IFA no grass—calculations based on M3 crop map, excluding grass crops and IFA mineral fertilizer data.

Of SPAM‐based NUE, 65% lies within 10% of M3‐based NUE without including grass crops, as compared to the regional results where only the difference between M3‐based and SPAM‐based calculations for Central Asia and Russian Federation exceeds 10%. In Finland (FIN), crops belonging to the category “Other Cereals” highly influence the results, as they constitute 60% (M3) or 67% (SPAM) of the total harvested area. Higher production of these crops in SPAM leads to a higher N harvest, driving the NUE. Additionally, a high share of grass crops (31%) leads to more manure N and N deposition being added to the M3 N inputs, increasing the gap in NUE between the SPAM‐ and M3‐based results. In Portugal (PRT), M3 yields (24 t/ha) and SPAM yields (7 t/ha) differ substantially from each other when one of the most common crop types such as “Maize” is considered. For the crop type “Residuals,” M3 shows a yield of 6 t/ha, while SPAM shows a yield of 0.6 t/ha. These discrepancies can be explained by investigating the forage share of these crop categories. Forage maize makes up 90% of the total “Maize” production, and different forage crops such as turnips make up about 80% of the total “Residuals” production. In Cyprus (CYP), harvested areas and production per crop are very different in M3 and SPAM for most crop types. While SPAM, for example, allocates 35% of the total harvested area of Cyprus to “Other Cereals,” M3 does not allocate any area at all to this crop type.

In the case of Ireland (IRL), grass crops and the difference in cropland area attributed to Ireland could be responsible for the discrepancies. Discrepancies in New Zealand (NZL) stem from the combination of IFA fertilizer distribution and cropland area differences, leading to a more than three times higher manure input in the M3‐based calculations than in the SPAM‐based calculations. As New Zealand is also part of the IFA country group “ROW,” the derived mineral fertilizer application rate is very low which does not reflect the actual application rate (The World Bank Group, 2020). This difference disappears when fertilizer application is adapted to FAOSTAT values, as this input is much higher than the N input from manure (see supporting information for a more detailed elaboration). In Australia (AUS), higher SPAM yield for “Wheat” and higher N input from manure due to cropland area discrepancies explain the difference in NUE.

## 4 Discussion

### 4.1 Key Findings

**Figure 6:**
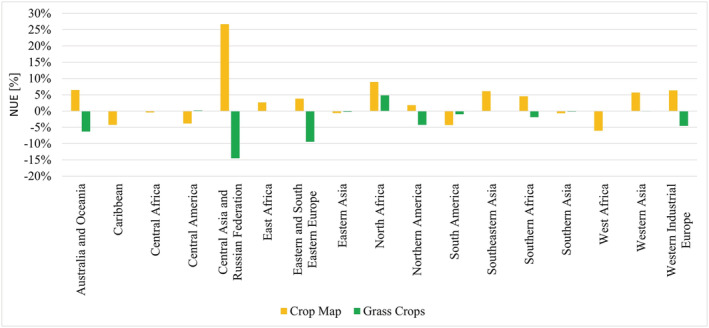
Differences in NUE and their dominating causes per region. To identify the causes different variations for the NUE calculations have been compared: Crop Map—difference between SPAM IFA and M3 IFA no grass; Grass Crops—difference between M3 IFA and M3 IFA no grass. SPAM IFA—calculations based on SPAM crop map and IFA mineral fertilizer data; M3 IFA no grass—calculations based on M3 crop map excluding grass crops and IFA mineral fertilizer data; M3 IFA—calculations based on M3 crop map including grass crops and IFA mineral fertilizer data.

Crop maps provide an opportunity to spatially distribute statistical data, thus enabling a look not only at the sub‐regional level but also at the influences of crop classification, allocation, distribution, and crop‐type‐specific characteristics such as yield. When calculating NUE and N surplus on two different crop maps, we noted that there are different reasons for discrepancies between maps, and those again differ between regions. We identified the inclusion of grass crops and crop map‐specific characteristics such as crop distribution, yield, and cropland area extent as the causes of certain discrepancies. Figure 6 shows which component of the respective NUE most impacts the outcomes per region, summarizing the results described in the previous section.

Including grass crops in the calculation influences the NUE results for all regions in which they are cultivated. When grass crops are included, NUE is generally lower due to increased N input, with the exception of North Africa where the high yield of clover leads to a higher NUE when grass crops are included; the same is true for high‐yielding alfalfa crops in Central America and Western Asia and for vetches in East Africa. The difference in crop maps can be identified as the main cause of discrepancies in Central Asia and the Russian Federation, Australia and Oceania, and North Africa. Although an update of harvested areas and production of grass and forage crops for the year 2010 was not possible due to a lack of data, these crop types play quite an important role in NUE calculation for some regions. Further research on this topic could help to make results more robust.

We also discovered small‐scale influences like the impact of olives in Southern Spain due to their categorization as “Other Oilseeds” in the SPAM‐based NUE calculation and the impact of high‐yielding clover in Egypt when grass crops are included. Sub‐regional discrepancies in crop distribution were found in India. Additionally, differences in cropland allocation were found in North America, Ireland, and Northwestern Russia, where there are many areas with only M3 cropland, whereas in Central Africa, Indonesia, Madagascar, and Papua New Guinea, M3 and SPAM allocate cropland to completely different areas (Table 3).

**Table 3:** Summary of Main Discrepancies and Causes Found Between SPAM‐ and M3‐Based NUE Calculations

	Cause	Explanation
Central Asia and Russian Federation	Crop Map & Grass Crops	Yield difference in “Wheat” (SPAM/M3: 1.3) and “Other Cereals” (SPAM/M3: 1.7) that are increased when N harvest is calculated (SPAM/M3: 1.3 & 1.9)
		More N input in M3 due to larger cropland area (grass crops)—M3: 4.4 Tg N, SPAM: 3.7 Tg N
North Africa	Crop Map/Crop Categorization	More N input in M3‐based NUE due to larger cropland area—M3: 2.6 Tg N, SPAM: 2.2 Tg
		SPAM inclusion of olives in “Other Oilseeds” leads to higher SPAM‐based N harvest—M3: 75 kt N, SPAM: 202 kt N
Australia and Oceania	Crop Map	Yield difference in “Wheat” and “Other Cereals”
		More N input in M3 due to higher production of “Residuals” leading to more BNF (M3: 72 kt N, SPAM: 60 kt N). More N input in M3 due to larger cropland area (grass crops)
Southern Spain	Crop Categorization	SPAM inclusion of olives in “Other Oilseeds” leads to higher SPAM‐based N harvest
India	Crop Distribution	Sub‐regional crop distribution differences between SPAM and M3
North America, Ireland, Russia	Crop/Grass Distribution	Many areas with only M3 cropland
Central Africa, Indonesia, Madagascar, Papua New Guinea	Cropland Allocation	M3 and SPAM allocate cropland to very different areas

### 4.2 Comparisons With Other Studies

To gain an impression of how our calculated values fit to the literature, we compared them to several other studies. The global totals for mineral fertilizer, manure N, N deposition, BNF, and harvested N are well in agreement with other literature and model values (FAO, 2019d; Fowler et al., 2013; IIASA AIR Group, 2018; Zhang et al., 2017) which is not surprising, as similar sources were used. The global NUE based on SPAM, 47%, or M3, 44%, is also close to the values calculated by Lassaletta et al. (2014), 47% and Bouwman et al. (2017), 49%. For a better idea of how our calculated values fit to other results on a country level, we compared the variations in M3‐based country NUE calculation and SPAM‐based country NUEs, discussed previously, with global country data from Lassaletta et al. (2014), European country data from Leip et al. (2011), and, where available, country data from Bouwman et al. (2017). A detailed table is presented in supporting information Data Set S2.

NUEs found by Lassaletta et al. (2014) and Leip et al. (2011) can differ substantially from each other for some countries. This could be explained by the use by Leip et al. (2011) of the CAPRI model, which allocates mineral fertilizer and manure N according to the crops' need for N as opposed to Lassaletta et al. (2014) who distribute all manure N ready for application on cropland and subtract a share of mineral fertilizer applied to grassland before considering it in their calculation. The M3 data with FAOSTAT fertilizer use, with fertilizer application to pasture subtracted, fits best to the data from Lassaletta et al. (2014): this can be explained by Lassaletta et al. (2014) using similar data such as, for example, FAOSTAT fertilizer and BNF rates from Herridge et al. (2008).

Discrepancies between our calculations (M3‐based and SPAM‐based results adjusted using FAOSTAT fertilizer for better comparability) and other sources can be traced back to different causes. A high discrepancy between the NUE from Lassaletta et al. (2014), 9%, and the M3‐ and SPAM‐based NUE in Malaysia (MYS), 39% and 39%, respectively, is rooted in the production of oil palm. Harvested area from oil palm cultivation makes up 65% (SPAM) to 69% (M3) of the total harvested area and 93% (M3 and SPAM) of total production, and therefore, of all crop types, is the biggest driver of NUE. As Lassaletta et al. (2014) do not assign any N harvest to oil palm, their NUE remains very small compared to M3 and SPAM. This difference in N content derives from our update of M3 yields to fit the FAOSTAT value for palm fruit, which is very similar to the SPAM yield and has an N content of 0.3%, whereas Lassaletta et al. (2014) seem only to have considered the oil component, without N content being assigned to it (Donough et al., 2016). With regard to Argentina (ARG), Lassaletta et al. (2014) used a lower BNF rate for soybeans, which are the dominant crop type in Argentina, leading to a higher NUE (105%), compared to our calculations (86% M3‐based and 79% SPAM‐based).

### 4.3 Proposed Way Forward

**Figure 7:**
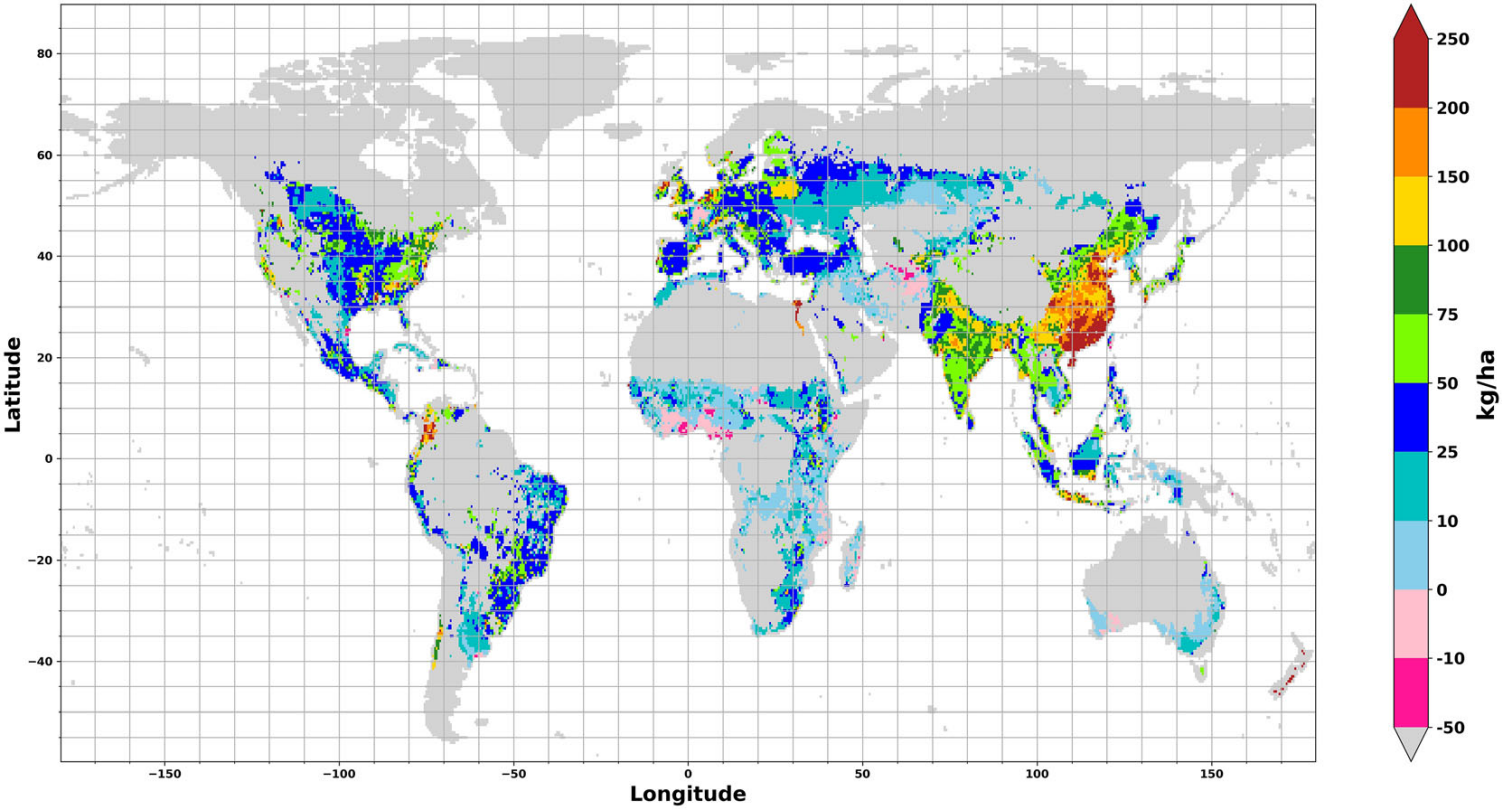
N surplus based on the M3 crop map with IFA fertilizer adjusted to FAOSTAT values.

Open scientific literature and publicly available information allowed us to acquire different relevant datasets required to develop gridded N indicators. To best represent N surplus, we identified a combination of the M3 crop map with fertilizer statistics based on IFA but adjusted to fit FAOSTAT country statistics as most appropriate. This suggestion is mainly based on the greater crop variety offered by the M3 crop map. That factor is important for calculating N indicators, as the aggregation of different crops into one category can, we found, lead to false results. For the specific purpose of this work, this combination proves to be more relevant than the benefits offered by SPAM. Due to rising N_2_O emissions on managed pastures (IPCC, 2020), the same combination of data sources will be useful for expanding calculations to include grassland area such as pastures and rangeland. Such calculations need a clear definition of the area covered in a crop map. This is provided in M3 as its cropland definition fits FAO's definitions of “permanent crops” and “arable land” (FAO, n.d.). Figure 7 presents our results for N surplus on a global grid using M3. It shows areas of high N surplus per hectare in China and India and in Northeastern North America, while countries in West Africa show N deficiencies.

## 5 Conclusions

The choice of an appropriate crop map is a key step toward improving the interpretation of the N cycle in agricultural soils. Parameters like N surplus and NUE values are highly sensitive to the choice of crop map, and a detailed understanding of data used is thus needed to allow an informed choice of product. This study offers the first detailed analysis of how discrepancies between crop maps affect the calculation of N indicators and it identifies their causes, highlighting opportunities for further research. Based on the knowledge gained from this study, a choice of crop map best fitting our needs was made, paving the way to expand the scope to also include grassland.

## Supporting information

Supporting Information S1

## Data Availability

The data that support the findings of this study are openly available in the supporting information and in the IIASA data repository (DARE) (at https://doi.org/10.22022/air/10‐2020.109).
