# Supplementary material for: Global Gridded Nitrogen Indicators: Influence of Crop Maps
Source: Global Biogeochem Cycles. 2020 Dec 7;34(12):e2020GB006634. doi: 10.1029/2020GB006634 (PMC7757259; doi:10.1029/2020GB006634)
Supplement: Supplementary file 1 — Supporting Information S1 [file GBC-34-e2020GB006634-s001.pdf]

[Global Biogeochemical Cycles]

Supporting Information for

[Global Gridded Nitrogen Indicators: Influence of Crop Maps]

[K. Kaltenegger[1], W. Winiwarter[1,2]]

[[1] International Institute for Applied Systems Analysis (IIASA), A-2361 Laxenburg, Austria

[2] Institute of Environmental Engineering, University of Zielona Góra, PL 65-417 Zielona Góra, Poland

Corresponding author: Katrin Kaltenegger (kalteneg@iiasa.ac.at)]

Contents of this file

Text S1 to S7
Figures S1 to S15
Tables S1 to S2

Introduction

This file contains detailed information on the calculation of all nitrogen (N) inputs and outputs to and from soil surfaces of two crop maps (SPAM and M3) which were used for the calculation of soil surface N budgets and NUEs. Most data used for these calculations was collected from several sources from autumn 2018 to spring 2019. Data was processed upon receival. This processing differed between the data types received and will be described in more detail throughout the following sections. The programming language "Python" was used for all calculations.

Additionally, complimentary results for soil surface N budget calculations using different N input variations for SPAM and M3 are shown in section S7.

Additional data related to this paper and datasets mentioned in this text can be accessed through the IIASA Data Repository (DARE): https://doi.org/10.22022/air/10-2020.109..

S1. Calculating Manure Nitrogen Excretion

To calculate nitrogen input to soil from manure excretion and management, data on total livestock numbers of cattle and small ruminants (sheep and goats) and the distribution of ruminant livestock production systems was taken from "Gridded Livestock of the World" (GLW) which was a project initiated by the Food and Agriculture Organization of the United Nations (FAO) and the Environmental Research Group Oxford (ERGO) (Robinson et al., 2014a, 2014b, 2014c, 2014d, 2014e, 2014f, 2014g). The grid containing total animal numbers is produced by combining several aspects. First a GIS map is developed containing sub-national statistical data on Livestock numbers per administrative unit (FAO-GAUL). Then a suitability mask containing information on elevation, slope gradient, protected areas and biophysical characteristics (elevations higher than 4750 m above sea level, areas with a slope gradient higher than 40%, protected areas and urban areas or areas permanently covered in snow or ice are excluded) was developed. Data was taken from several models (GTOPO30 model, WDPA and GLC2000). Additionally, a layer containing predictor variables such as length of plant growth (LPG), population density and travel times to areas with a population of more than 50000 people, temperature, precipitation, green- up, senescence was created as was a layer containing agro-ecological zones due to the circumstance that different predictor variables and different zones could have different implications for animal densities. Bootstrap technique (calculating inferences from the sample of a sample) and regression are used to calculate the final predictions for animal density. After this process, the outcomes are compared to FAO statistics and corrected if necessary.

The data was available as a 5' grid in tiff format and was converted to a 0.5-degree grid by summing up the respective 36 cells between 0.5-degree latitude and 0.5-degree longitude. The dataset using the dasymetric method was chosen, meaning that the distribution of livestock is based on population, vegetation and topographic information.

Manure nitrogen excretion for cattle and small ruminants was calculated using the beforehand calculated livestock grid and nitrogen excretion rates per GAINS (Greenhouse Gas – Air Pollution Interactions and Synergies) region, taken from the GAINS model (International Institute for Applied Systems Analysis AIR Group [IIASA AIR Group], 2018a) (1). Since nitrogen excretion is higher for dairy cattle, a differentiation was introduced between dairy cattle and other cattle by using a weighted average of milk cows per GAINS region calculated from FAOSTAT livestock data available per country since this differentiation was not included in the gridded data. The milk cow ratio was weighted using shares of manure nitrogen excretion of dairy cattle and other cattle per country (FAO, 2019e). The procedure to calculate the average of milk cows per GAINS regions was chosen for consistency reasons because data on milk yield influencing nitrogen excretion rates was later taken from GAINS and was only available for each GAINS region (IIASA AIR Group, 2018d).

$$N_{ExC} = \sum_{GAINS\ region\ i} LC * p_i * (p_D * r_{ExDi} + (1 - p_D) * r_{ExNDi}) \quad (1)$$

NExC… nitrogen excretion from cattle per grid cell
LC… livestock number in grid cell as calculated from GLW data
pi… percentage of cell area belonging to GAINS region i
pD… percentage of dairy cattle

rExDi… GAINS-region specific nitrogen excretion rate for dairy cattle according to GAINS (milk yield included)
rExNDi… GAINS-region specific nitrogen excretion rate for non-dairy cattle according to GAINS

In addition to the livestock data from GLW, data on livestock management systems (LMS) used for chicken and pigs was provided by an FAO employee who works on developing the Global Livestock Environmental Assessment Model (GLEAM). This was necessary to approximate the number of layers and other poultry as well as industrial and non-industrial pigs in GLW data to account for layers and industrial pigs having a higher nitrogen excretion rate. For the GLW dataset on global pig number the influence of Muslim population on pig densities was considered by assigning a zero-pig density to countries with a Muslim population higher than 50% and sub-national GLIMS data as well as FAOSTAT data indicating zero or no data for pigs.
A tiff file was received for each livestock management system per animal type providing information on the livestock numbers kept per system in the year 2006. This data was converted and combined to create one file for chicken livestock management systems and one file for pig livestock management systems in a 0.5-degree grid format containing the total number of livestock being held per system and per 0.5-degree cell as well as the percentage of animals being held per system. The percentages were then combined with the total livestock number for the year 2010 to approximate the number of livestock held per system for this reference year. Then, following the same procedure used to calculate ruminant total nitrogen manure excretion, nitrogen excretion rates per livestock management type were taken from the GAINS model for each GAINS region as can be seen in (2).

$$N_{ExM} = \sum_{GAINS\ region\ i} LC * p_i * \sum_{LMS\ j} p_j * r_{ji} \qquad (2)$$

NExM… nitrogen excretion from pigs or chicken in one grid cell
LC… livestock number in grid cell as calculated from GLW data
pi… percentage of cell area belonging to GAINS region i
pj… share of total livestock in LMS j
rji… GAINS-region and LMS specific nitrogen excretion rate according to GAINS

S2. Calculating Manure Nitrogen Application

When calculating manure nitrogen application, the gridded excretion as calculated before was combined with the gridded dataset on livestock production systems for ruminants made available by GLW and a gridded dataset on livestock production systems for monogastrics which was used for a previous GLEAM version (GLEAM 2) (FAO, 2010) provided by a FAO employee. These datasets do not show the real distribution of livestock production systems but rather a prediction of these systems. This prediction is based on Global Land Cover (GLC) land use data, population data, climate data, data on length of growing period and data on irrigated areas (Robinson et al., 2014a). Livestock production systems for ruminants are differentiated according to the definition introduced by Seré and Steinfeld (1996) into solely livestock systems and mixed farming systems with the difference being that in the latter more than 10 percent of the animal feed comes from crop by-products or stubble or “more than 10 percent of the total value production comes from non-livestock farming activities”. The mixed systems are divided into rainfed and irrigated systems. Each system is also divided according to the agroclimatic categories arid, humid and temperate which leads to overall nine different livestock production systems. Livestock production systems for poultry are divided into three categories: broilers, layers and backyard

poultry. Pig livestock production systems are also divided into three categories: industrial, medium and backyard pigs.
The data on livestock systems was available as a 0.5’ grid and was again converted to a 0.5-degree grid. Whereas the ruminant livestock system grid showed which livestock system was dominant in each grid cell, the monogastric livestock system grid displayed the total number of animals held by each livestock system.

$$N_{Appl} = \sum_{GAINS\,region\,i} \sum_{LMS\,j} N_{Ex} * p_i * p_{mmj} * p_j + (1 - p_{lossj}) \quad (3)$$

NAppl… nitrogen application for a livestock type in one grid cell
NEx… nitrogen excretion from a livestock type in one grid cell
pi… percentage of cell area belonging to GAINS region i
pmmj… percentage of excreted manure that is being manage per LMS
pj… share of LMS in grid cell
plossj… percentage of manure nitrogen lost during manure management in LMS

This distribution of livestock production systems was then combined with the livestock data calculated before as well as the percentages of manure handled per livestock production system and the percentage of manure lost through volatilization and leaching during the storage in the respective system according to (3). Data on the amount of manure being managed and manure nitrogen losses in the respective livestock production systems was taken from Herrero et al. (2013) and follows IPCC guidelines as well as expert opinions and measurements. This data is divided into five world regions – Africa, Asia, Europe, North America and Latin America which are further on referred to as “Herrero regions”.

To calculate nitrogen input from manure on cropland, only manure managed and applied was included due to the assumption of unmanaged animal droppings being excreted on pasture- and rangeland. Managed manure on cropland was calculated using different fractions for different countries following the procedure described in Liu et al. (2013) that allows to exclude the amount of manure applied to pastureland. As no differentiation between different US states was made in our calculations, we used the average of 87% of manure going to cropland. For developing countries, it was assumed that 90% of manure is applied to cropland, as described by Smil (1999). Following Menzi et al. (1998), different shares of manure application to cropland were used for different European countries, but no differentiation between animal types was made. For Canada and European countries not mentioned in Menzi et al. (1998), an average of 66% of manure was assumed to be recycled to cropland. For the remaining countries that were not included in any of the beforementioned studies, a share of 50% of manure application to cropland was assumed.

S3. Calculating Mineral Fertilizer Application

Nitrogen input from mineral fertilizer was calculated by combining data on harvested area for 175 different crops by Monfreda et al. (2008) (M3 crop map) or respectively data on harvested area for 42 different crop types from SPAM (International Food Policy Research Institute, 2019; You et al., 2014) with statistics by the international fertilizer agency (IFA) for the year 2010 (Heffer, 2013). The data provided by IFA for 2010 was available for 28 different countries or country categories and 14 different crop categories:

Countries:
ROW – rest of world, Argentina, Australia, EU-27, Brazil, Chile, China, Egypt, Indonesia, India, Iran, Japan, Morocco, Mexico, Malaysia, Pakistan, Philippines, Russia, Thailand, Turkey, USA, Uzbekistan, Vietnam, South Africa, Canada, Belarus, Ukraine

Crop Categories:
Fruits, Roots and Tubers, Oil Palm, Residual, Oth(er) Oilseeds, Vegetables, Oth Cereals, Fibre Crops, Sugar Crops, Soybean, Wheat, Rice, Maize, Oth Crops

Due to the existence of grass crops (alfalfa, clover, vetches, mixed grass, fornes (forage not elsewhere specified) and grass nes (grass not elsewhere specified)) in the M3 crop map, the IFA category "Other Crops" from 2010 was divided into "Residual crops" and "Grass crops" which were only introduced in 2014 (Heffer et al., 2017). To do so, a factor describing the share of 'Grass Crops' in 'Other Crops' was derived from the 2014 data.

For the distribution of mineral fertilizer on cropland and pastureland, first, harvested areas were added together to fit the IFA crop categorization. In a next step, IFA fertilization rates per harvested area, crop category and country were calculated and then distributed accordingly on the grid cells by multiplying the rates with the updated harvested area values for 2010 per grid cell.

Although the global total of mineral fertilizer use found in FAOSTAT data matched the global total found in IFA data quite well, there were significant differences between regional data which again differed between M3 and SPAM. Due to these differences, each grid cell was updated so that the sum of all grid cells belonging to a country would match the FAOSTAT data (FAO, 2019a).

FAOSTAT data is described to include mineral fertilizer used for pastures and aquacultures but only shows the sum of mineral fertilizer applied to all crops per country and not the amount applied to different crop types. While aquacultures do not contribute significantly to the total fertilizer use of any country, pastures can make a difference in countries like Ireland and New Zealand.

For the update of each grid cell to fit FAOSTAT data, first, all mineral fertilization data for each country was summed up. For each country a multiplication factor displaying the difference between the IFA and FAOSTAT data was calculated and then each grid cell belonging to a certain country was multiplied with the corresponding factor to fit FAOSTAT country sums. For the subtraction of the amount of mineral fertilizer applied to pastureland, information was taken from Lassaletta et al. (2014) provided in the supplementary material.

We compared M3 and SPAM calculations with IFA fertilizer to M3 and SPAM calculations using the FAOSTAT adjusted fertilizer use to identify areas where the crop and region-specific allocation of IFA data leads to an over- or underestimation of mineral fertilizer use (see Figure S1 for regional differences in mineral fertilizer application between these two sources).

Areas most affected are Central, West and East Africa, while for Central Asia and the Russian Federation and Western Industrial Europe it depends whether one compares the mineral fertilizer taken from IFA distributed on M3 to the same fertilizer data distributed on SPAM with FAOSTAT data. The difference spotted in African regions, is due to the fact that most African countries can

be found in the IFA country category ‘ROW’ (rest of the world) together with Central American and some Asian countries which have an up to tenfold higher fertilizer application rates (World Bank, 2020). However, due to the distribution procedure, every country in a country category gets assigned the same fertilizer application rate. Due to differences in crop distribution and because M3 includes forage crops in the crop categories ‘Residual’ and ‘Maize’ which are not included in SPAM, fertilizer application rates can differ between M3 and SPAM as for example in Central Asia and the Russian Federation.

Mineral fertilizer application to pastures also influences these differences as it is subtracted from the FAOSTAT data (see Methods). This influence, which can again differ between the SPAM and M3 based calculations, can for example be observed when looking at Western Industrial Europe, where about 20% of mineral fertilizer is applied to pastures. Using M3 based calculation for this region, IFA fertilizer application is increased by only 10% to fit the FAOSTAT number leading to a higher NUE with FAOSTAT numbers due to lower N input. However, as IFA fertilizer application in the SPAM based calculation is increased by over 35% to fit FAOSTAT, exceeding the amount that is subtracted from the FAOSTAT number by over 10%, the NUE of the IFA based calculation is higher.

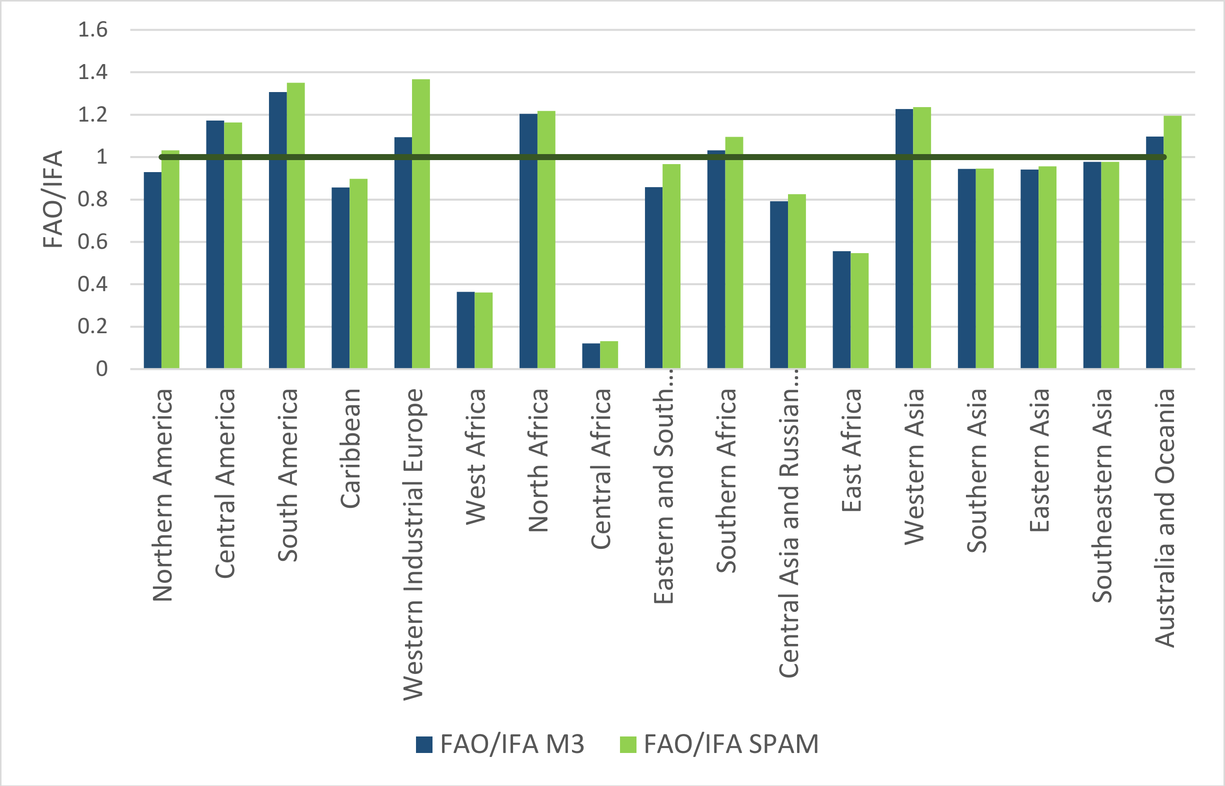

Figure S1 Ratio between FAOSTAT (amount applied to permanent pastures subtracted) and IFA mineral fertilizer when distributed using M3 harvested areas and when using SPAM harvested areas for distribution per world region.

Volatilization was calculated using GAINS factors for region specific fractions of Urea and non-Urea in total mineral fertilizer use as well as region specific fractions of flooded rice as these factors impact the amount of NH_3 and N_2O that volatilizes (International Institute for Applied Systems Analysis AIR Group, 2018b, 2018c) (4). Region specific emission factors for NH_3 and N_2O

were also taken from GAINS. Due to rice having different N_2O emissions depending on its cultivation, a percentage of harvested areas of rice in total harvested area was multiplied with the rice specific factors (5).

$$lossNH3_{c,i} = PercU \times PercVolNH3\_U + (1 - PercU) \times PercVolNH3\_NU \quad (4)$$

$$lossN2O_{c,i} = 0.02 \times (1 - percRice) + (PercNFlood \times 0.02 + PercFlood \times 0.011) \times percRice \quad (5)$$

$PercU$… region specific percentage of Urea in mineral fertilizer
$PercVolNH3\_U$… region specific percentage of NH_3 volatilization from Urea
$PercVolNH3\_NU$ … region specific percentage of NH_3 volatilization from non-Urea
$percRice$ … percentage of harvested areas of rice in total harvested area
$PercNFlood$ … percentage of non-flooded rice
$PercFlood$ … percentage of flooded rice
0.011 … N_2O emission factor for flooded rice
0.02 … N_2O emission factor for non-flooded rice

The amount of N volatilization was summed up for all countries in all cells and then subtracted from the total mineral fertilizer application.

S4. Biological Nitrogen Fixation

To calculate biological N fixation (BNF), data by Herridge et al. (2008) was used for crops and data by Smil (1999) was used for grass crops in the M3 calculation. Herridge et al. (2008) calculated BNF by combining data on yield areas of legumes and cereals provided by FAOSTAT. Below ground N resulting from BNF was considered in their calculations as well as the difference between symbiotic BNF and BNF by free living bacteria. All values are listed in Table S1. and Table S2.

Table S1. Nitrogen fixation rates per crop type from Herridge et al. (2008) for the year 2005 using SPAM harvested areas

Agent	Agricultural System	SPAM Harvested Area [ha]	Rate of N2 fixation [kgN/ha/year]	Crop N fixed [Tg/year]
Legume-rhizobia	Common bean	29,287,465.24	19.80	0.58
Legume-rhizobia	Cowpea	10,571,177.90	21.76	0.23
Legume-rhizobia	Chickpea	12,279,807.01	48.86	0.60
Legume-rhizobia	Lentil	4,055,468.77	51.78	0.21
Legume-rhizobia	Other Pulses	20,401,553.32	23.04	0.47
Legume-rhizobia	Groundnut	25,128,088.61	81.98	2.06
Legume-rhizobia	Soybean USA	30,541,195.00	187.94	5.74
Legume-rhizobia	Soybean BRA	22,944,459.00	200.92	4.61
Legume-rhizobia	Soybean ARG	18,096,735.00	190.09	3.44
Legume-rhizobia	Soybean CHN	8,493,378.00	111,85	0.95
Legume-rhizobia	Soybean ROW	21,864,881.00	172.70	3.73
Azolla-cyanobacteria; Cyanobacteria;	Rice	160,727,200.70	31.11	5.00

Endophytic, associative and free-living bacteria				
Endophytic, associative and free-living bacteria	Sugar cane	24,335,786.53	20.55	0.50
Endophytic, associative and free-living bacteria	Crop lands other than used for legumes and rice	912,439,254.80	3.29	3.00

Note: BNF rats for soybeans were calculated from the average of BNF rates assigned to soybeans in USA, Brazil, China and Argentina.

Table S2. Nitrogen fixation rates per crop type from Herridge et al. (2008) for the year 2005 using M3 harvested areas and Smil (1999) for grass crops (alfalfa, vetches, fornes, clover, grassnes and mixedgrass)

Agent	Agricultural System	Harvested Area [ha]	Rate of N2 fixation [kgN/ha/year]	Crop N fixed [Tg/year]
Legume-rhizobia	Common bean	30,775,118.50	18.80	0.58
Legume-rhizobia	Cowpea	11,500,187.43	20.00	0.23
Legume-rhizobia	Chickpea	11,898,128.55	50.40	0.60
Legume-rhizobia	Pea	6,577,724.82	86.70	0.57
Legume-rhizobia	Lentil	4,289,270.57	49.00	0.21
Legume-rhizobia	Fababean (Broadbean)	2,529,260.45	115.00	0.29
Legume-rhizobia	Other Pulses	17,625,270.70	26.70	0.47
Legume-rhizobia	Groundnut	26,603,423.75	77.40	2.06
Legume-rhizobia	Soybean USA	31,003,300.00	191.33	5.74
Legume-rhizobia	Soybean BRA	23,327,296.00	201.31	4.61
Legume-rhizobia	Soybean ARG	18,130,800.00	245.71	3.44
Legume-rhizobia	Soybean CHN	8,515,750.00	98.96	0.95
Legume-rhizobia	Soybean ROW	21,818,126.05	171.00	3.73
Legume-rhizobia	Alfalfa	20,083,540.50	200.00	4.02
Legume-rhizobia	Clover	2,164,545.76	150.00	0.32
Legume-rhizobia	Vetch, Forage (fornes)	18,055,536.00	100.00	1.80
Legume-rhizobia	Grasses (mixedgrass, grassnes)	70,576,598.78	80.00	5.65
Azolla-cyanobacteria; Cyanobacteria; Endophytic, associative and free-living bacteria	Rice	161,685,069.70	30.90	5.00
Endophytic, associative and free-living bacteria	Sugar cane	23,568,440.55	21.20	0.50

Endophytic, associative and free-living bacteria	Crop lands other than used for legumes and rice	922,338,909.90	3.25	3.00

Note: BNF rats for soybeans were calculated from the average of BNF rates assigned to soybeans in USA, Brazil, China and Argentina.

While the fact that capacity for N fixation changes with soil and plant-growth conditions for legumes is discussed by Herridge et al. (2008) but found to be beyond their scope of consideration, this differentiation is only made for soybeans as they are responsible for most of the nitrogen fixed by legumes. US soil used for soybean production is fertile with moderate to high concentration of plant-available nitrogen which leads to a high N fixation rate. In Brazil, rhizobial inoculation, a low fertilizer usage and the practice of no-till farming lead to a high N_2 fixation rate. The same is true for Argentina, whereas in China fertilizer use and residual minerals in the soil lead to a lower N fixation rate. Herridge et al. (2008) do not discuss BNF rates for soybeans outside US, Argentina, China and Brazil. However, because we found soybean production in other parts of the world too, we calculated an average BNF rate from the BNF rates for soybean production in these four countries.

For the calculations used for this work, annual N fixation per crop type was taken from Herridge et al. (2008) and divided by the amount of harvested area for each crop category (summed up from each crop map to fit the types listed by Herridge et al. (2008)) from the respective crop map. The derived BNF rate was then used to distribute the annual amount fixed according to Herridge et al. (2008) on the crop map areas. We chose to take the annual amount of N fixed and not the BNF rates given by Herridge et al. (2008) because the BNF rate relates to total physical area, which was not available per crop type using M3.

As Herridge et al. (2008) only presented one BNF rate for pasture and leguminous crops and did not differentiate between leguminous pasture crops such as alfalfa and clover and mixed pasture crops such as mixed grass, we decided to take values from Smil (1999) for the M3 calculation instead. Smil (1999) differentiates forages into alfalfa, clover and other forages, giving ranges of possible BNF rates for each of these crop types. From this range (mean, upper and lower boundary) we always took the mean value except for mixed grass and grassnes where we took the lower boundary allowing for a differentiation between leguminous forage crops such as vetches and fornes (forage not elsewhere specified) and the grasses within the category ‘other forages’ given by Smil (1999). We are aware that BNF fixed by crop categories such as mixedgrass and grassnes comes with a large uncertainty because the share of leguminous crops in this mix and the contribution of free-living bacteria are hard to estimate. Using the BNF rates given by Smil (1999) for all grass crops in M3 results in a global total of 11.8 Tg/yr which is slightly below the range of 12-25 Tg/yr given by Herridge et al. (2008) for pasture and fodder crops.

S5. Nitrogen Harvest

To calculate the nitrogen in harvest, information on total production per crop was taken M3 or SPAM. Since the M3 data was representative for the year 2000, each grid cell was updated using FAOSTAT production data for 2010 using the same procedure as for the update of harvested areas (see Methodology section in main script). Information on nitrogen content found in each crop type was taken from a document provided by the Expert Panel on Nitrogen Budgets (EPNB) (Winiwarter and the Expert Panel on Nitrogen Budgets, 2016) and was complimented by other literature when no information was available (e.g Donough et al., 2016 for oil palm, Pushparajah,

1969, Jurasek et al., 1994 and Lindenmayer et al., 1994 for gum). Data provided by Geisseler (2016) was used for comparison only since the data was only collected in California. The nitrogen content from all sources was converted to percentage of nitrogen per crop production unit and was then multiplied with the production information for every crop cell. FAOSTAT production for cereals contain only data on dry grains, which was respected when looking for nitrogen contents (FAO, n.d.b). For forage crops, data on nitrogen content including considerations of moisture at harvest provided by Lassaletta et al. (2014) was taken. For grass crops, N content taken from Winiwarter and the Expert Panel on Nitrogen Budgets (2016) was multiplied with 0.2, representing a dry matter content of 20% at harvest (Turano et al. 2016).

Data Set s01. Nitrogen content per crop for all M3 crops and SPAM crop types.

S6. Nitrogen Deposition

To calculate total nitrogen deposition, data from the Chemistry-Climate Model Initiative (CCMI) provided by Tian et al. (2018) was taken. This data was available as netCDF files (NHx and NOy separately) containing matrices with entries for 0.5x0.5degree cells for each month from 1860 to 2014. The average of the 12 months of 2010 was calculated for each grid cell. Total nitrogen deposition calculated for 2010 is very similar to the findings of Lamarque et al. (2013) for the year 2000.

Since deposition per grid cell included all land found in this grid cell, maps for SPAM and M3 containing fractions of cropland per grid cell were produced to only include the corresponding share of nitrogen deposition. The distribution and amount of cropland areas for the balances based on the M3 crop map was taken from Ramankutty et al. (2008) since the distribution of crops on the M3 map is based on the cropland distribution described by Ramankutty et al. (2008). However, since the data provided by Ramankutty et al. (2008) was from the year 2000, it was updated to 2010 using FAO data (FAO, 2019d). Total areas were updated using FAOSTAT country data and distributing it the same way harvested areas and yields were updated (see paper (3)).

S7. Complimentary Results and Details Used for Analysis

To arrive at the results presented in the manuscript, all budget terms and especially their crop map dependent variation for each region but also country and crop category was analyzed as described below.

Cropland Area

Discrepancies in cropland area found in M3 and SPAM is high for most regions (Figure S3). This can mostly be explained by M3 including grass crop areas while SPAM excludes them.

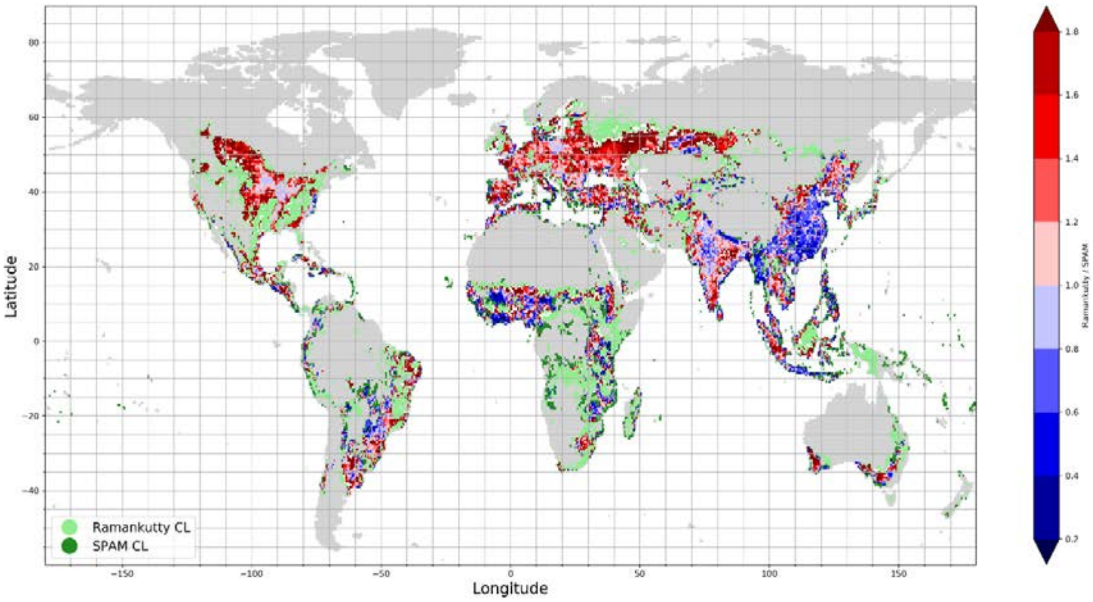

Figure S2. Comparison of physical cropland area between SPAM and M3

Manure N

Manure N application discrepancies mainly depend on cropland allocation in each map. Manure N is derived from FAO GLW (gridded livestock of the world). Differences between M3 and SPAM manure N application depend on how well grid cells showing cropland in the respective crop map fit to the livestock distribution from FAO GLW.

Manure that is managed and recycled to cropland is filtered to include only cells on which cropland bigger than 5% of the land area is found to exclude outliers. As can be seen in Figure S3 and S4, not all cells where manure N can be found according to FAO GLW contain more than 5% cropland. However, as can be seen in Figure S5, areas excluded are areas with very low manure N application. See s4 for a detailed country comparison.

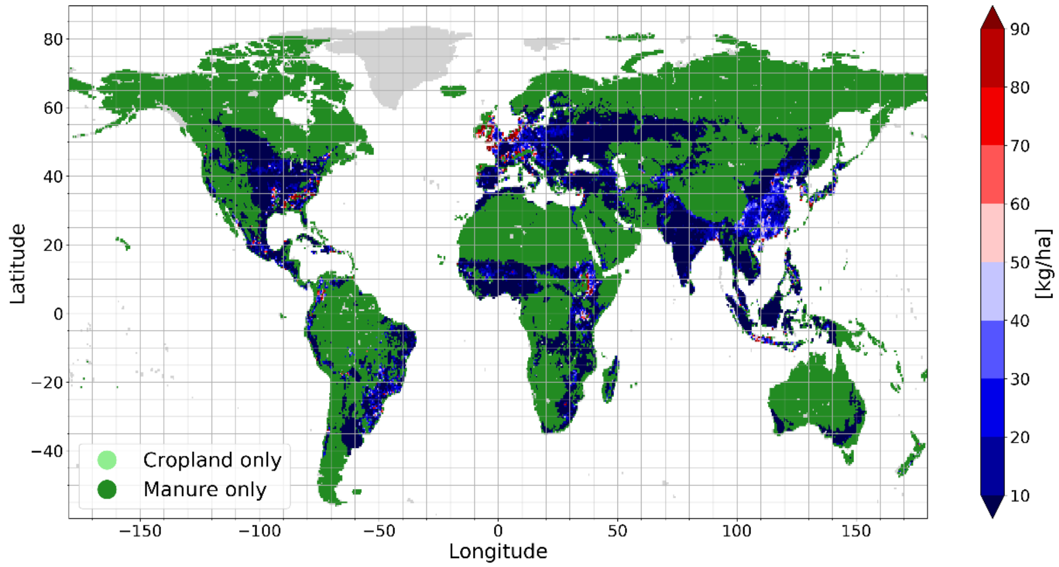

Figure S3 Kilogram Manure N per hectare M3 cropland. Cells where only manure N but no cropland area (>5% of total area) can be found are coloured dark green

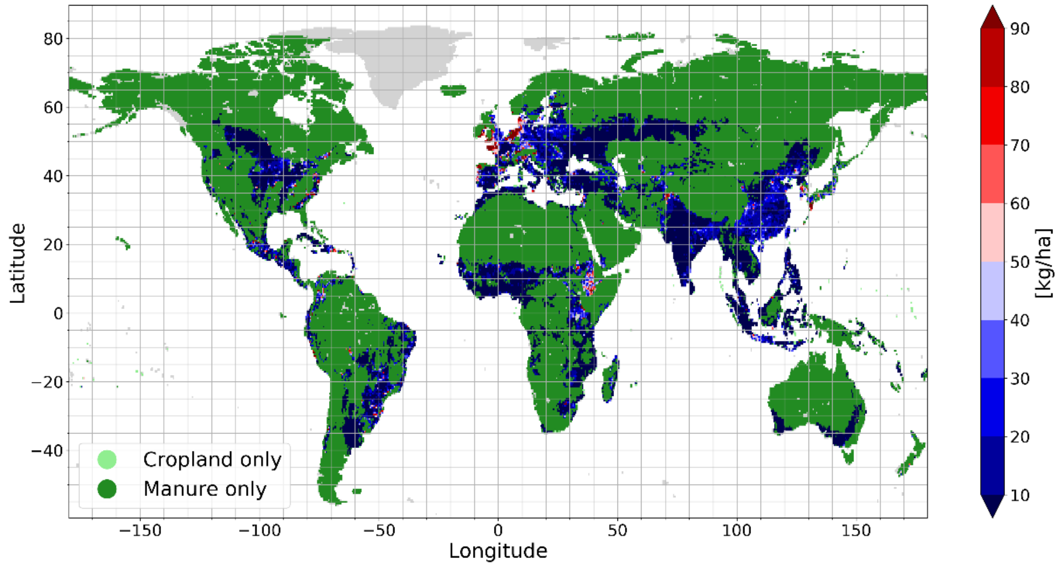

Figure S4 Kilogram Manure N per hectare SPAM cropland. Cells where only manure N but no cropland area (>5% of total area) can be found are coloured dark green

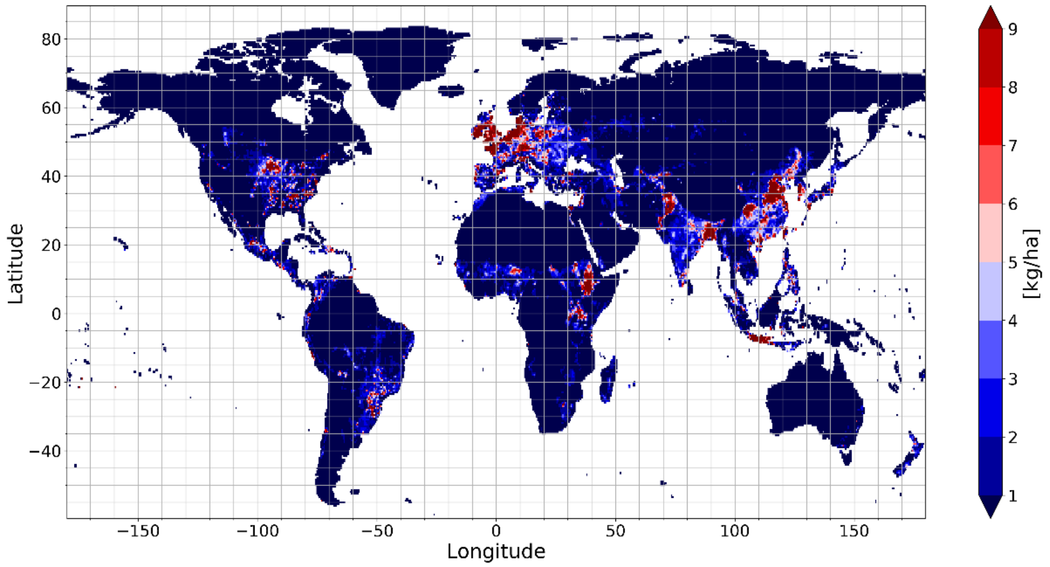

Figure S5 Kilogram manure N per hectare land area applied to cropland

N deposition

Discrepancies in N deposition follow the discrepancies of cropland area found in SPAM and M3. This was to be expected as the share of N deposition allocated to a country depends on that country's share of cropland area (see 'Methodology'). However, as manure N, cropland allocation also effects these results as cells where N deposition is shown are excluded when the respective crop map allocates less than 5% cropland to this cell.

Harvested Area

Harvested areas taken from M3 and SPAM are globally very similar. On a regional basis, a higher discrepancy of harvested area can be found in Western Industrial Europe (Figure S6). This discrepancy stems from the crop categories 'Residuals' and 'Maize' where M3 finds larger harvested areas than SPAM. Looking closer at these two categories, this difference can most likely be explained by forage crops which can be found in both crop categories in M3 but are not explicitly considered in SPAM. However, it was decided not to exclude them, as it was stated by the SPAM team (U. Wood-Sichra, personal information 2019) that some forage crops are included for various categories such as maize, barley and other pulses.

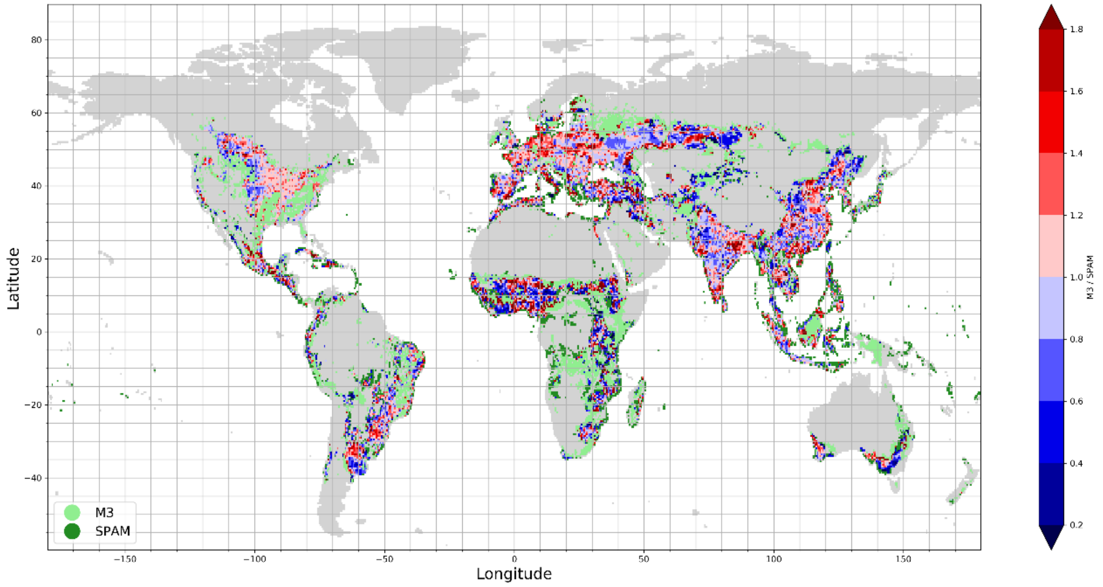

Figure S6 Ratio of M3 to SPAM for harvested area

<u>BNF</u>

While the ratio of SPAM to M3 for BNF is very similar, differences caused by differing crop composition of the categories because visible especially in Eastern and South Eastern Europe, Western Industrial Europe and North Africa. These differences in crop category composition mostly concern Residuals, where all pulses are included and M3 generally shows higher production values for this category. The finer crop resolution for this crop category found in M3, allows a more detailed allocation of BNF (see supplementary material S4 (Table S2 and Table S3)). This means that using M3, rather high BNF rates (e.g. 88 kg/ha for peas and 115 kg/ha for Fababeans) are assigned to crops that are not explicitly mentioned in SPAM but are expected to be included in the crop category other pulses, which is assigned an average BNF rate of 23 kg/ha.

<u>Crop production</u>

More regional discrepancies and a slight global discrepancy can be found when looking at crop production taken from M3 and SPAM. Regions showing the biggest discrepancies between the two crop maps are the Caribbean, Eastern and Southeastern Europe, Southeastern Asia and Northern America (Figure S7). While the discrepancies in the Caribbean are mainly caused by a differing production of fruit, the discrepancies in Eastern and Southeastern Europe and Northern America can be explained by differing production numbers of Maize. In Southeastern Asia, M3 assumes higher production for all crop types than SPAM.

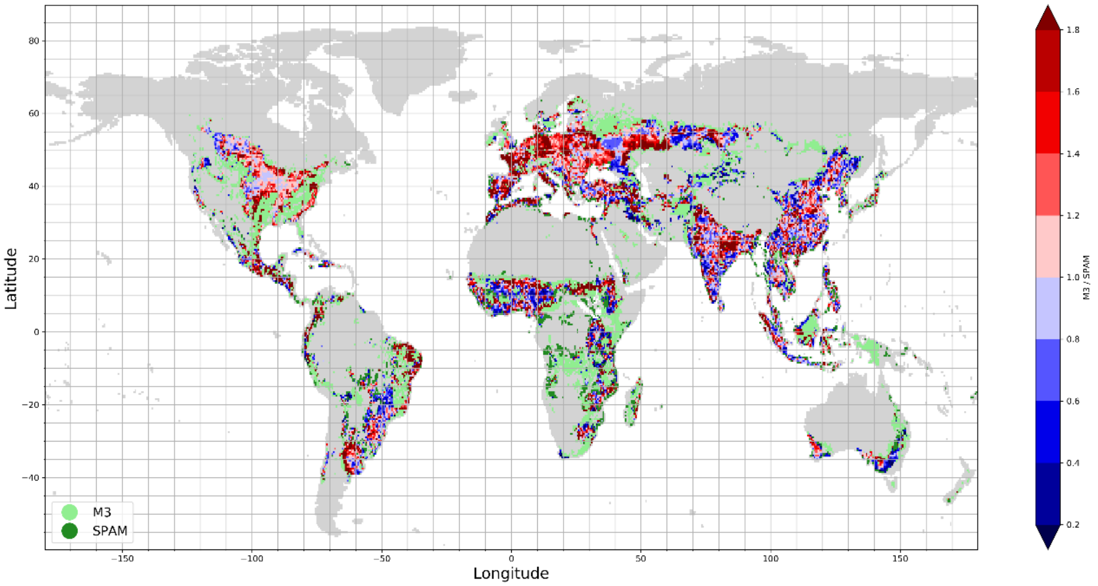

Figure S7 Ratio of M3 to SPAM for production

<u>N harvest</u>

The discrepancies in N harvest also display the effect the crop composition of a crop category has on the results. Looking at Eastern and South Eastern Europe, the crop category Residuals shows high production discrepancies between the two maps stemming from a high forage crop production assumed in M3. However, these discrepancies are reduced when looking at N harvested in this crop category, as forage crops have a very low N content.

In West Africa, a different crop distribution in the maps lead to low discrepancies in production as for some crop category, production is higher in M3 and for others it is higher in SPAM. However, as M3 shows more production in categories such as residuals, other oilseeds and other cereals which are assigned a higher N content, West Africa shows a discrepancy between M3 and SPAM for N harvest. This is similar for Central America, where the crop categories concerned are Residuals, Soybean and Maize and Central Asia and Russian Federation, where the crop categories leading to higher N harvest discrepancies are Wheat and Other Cereals.

In North Africa, discrepancies in N harvest between the two crop maps are related to the crop category other oilseeds and especially to Olives. This can be explained M3 showing less production of other oilseeds and additionally having a higher crop resolution within this category, enabling the assignment of different N content to each crop. As olives have a very low N content but account for about two thirds of the production of other oilseeds, they reduce the overall N harvest in the M3 based calculations compared to the SPAM N harvest that does not include Olives or other crops with an equally low N content.

The following figures compliment the results presented in the main script.

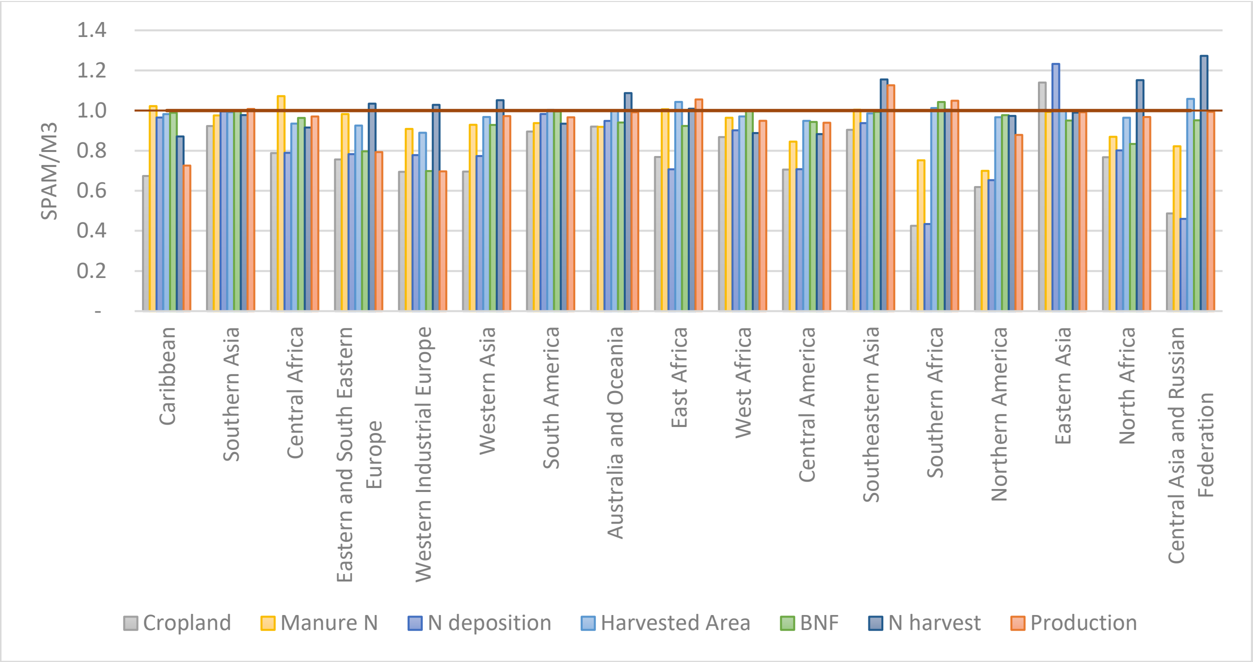

Figure S8. SPAM to M3 ratio for each world region and each N input as well as cropland, harvested area and production,

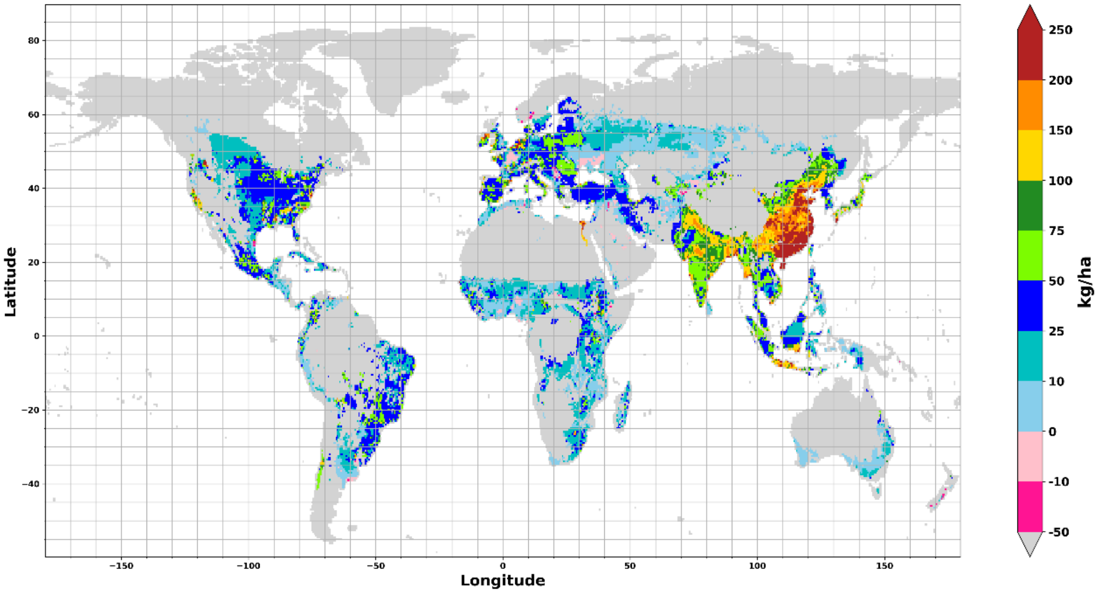

Figure S9. Soil Surface N budgets on M3 cropland without grass crops using IFA fertilizer with volatilization losses subtracted

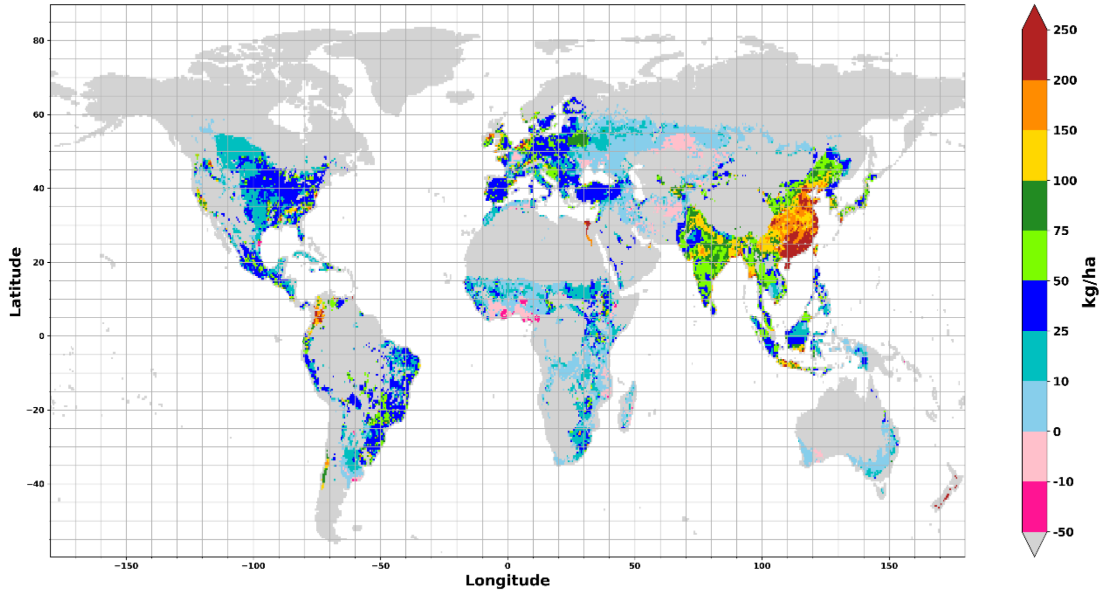

Figure S10. Soil Surface N budgets on M3 cropland without grass crops using FAO fertilizer with fraction on pastureland and volatilization losses subtracted

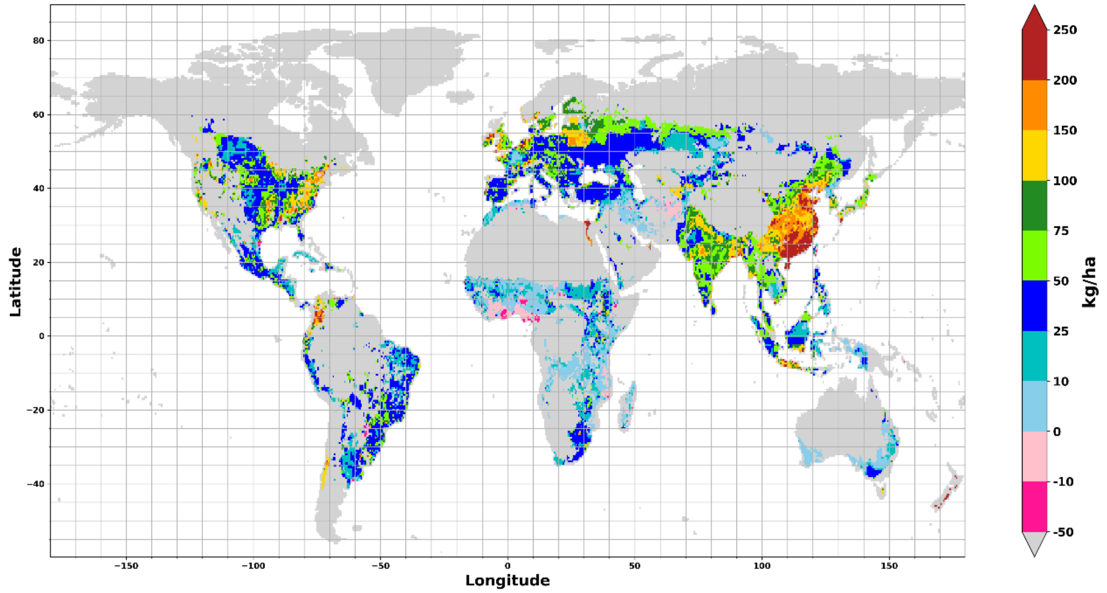

Figure S11. Soil Surface N budgets on M3 cropland with grass crops using FAO fertilizer with fraction on pastureland and volatilization losses subtracted and Herridge et al. (2008) BNF for grass crops

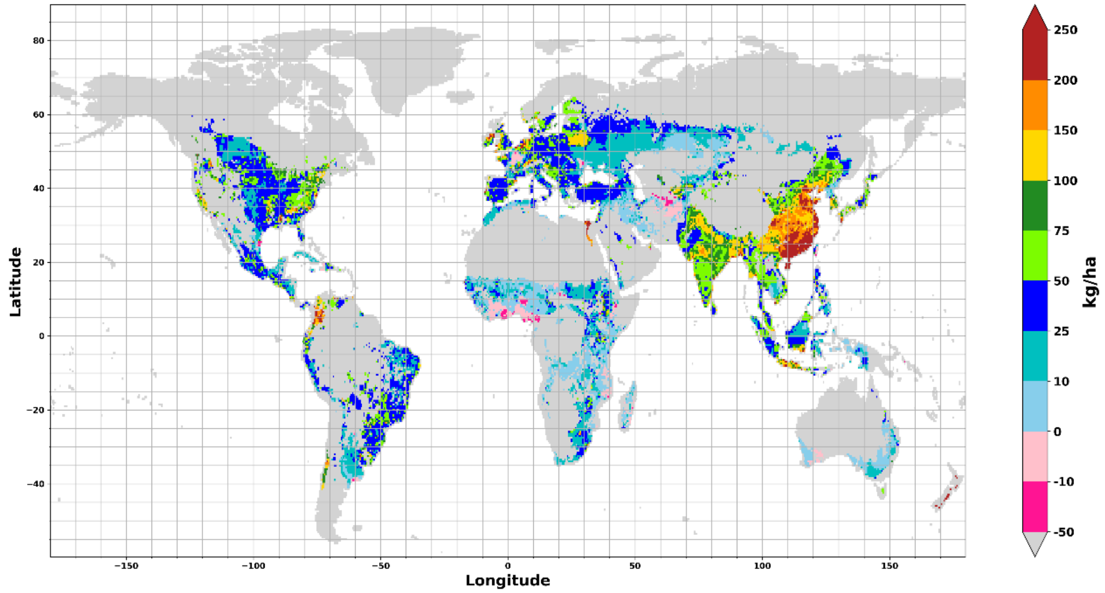

Figure S12. Soil Surface N budgets on M3 cropland with grass crops using FAO fertilizer with fraction on pastureland and volatilization losses subtracted and Smil (1999) BNF for grass crops

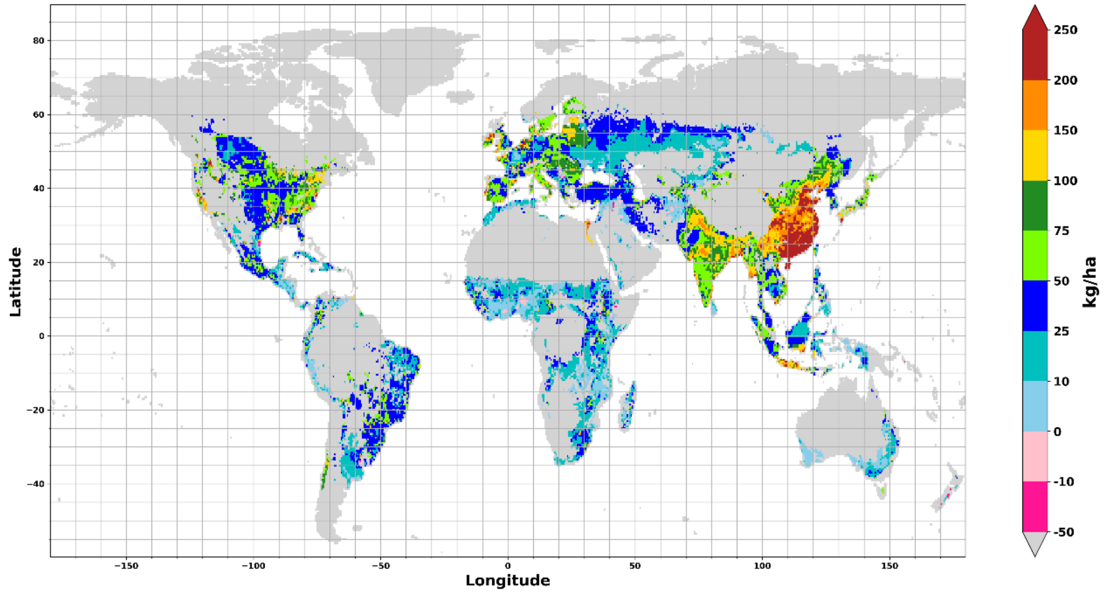

Figure S13. Soil Surface N budgets on M3 cropland with grass crops using IFA fertilizer with volatilization losses subtracted and Smil (1999) BNF for grass crops

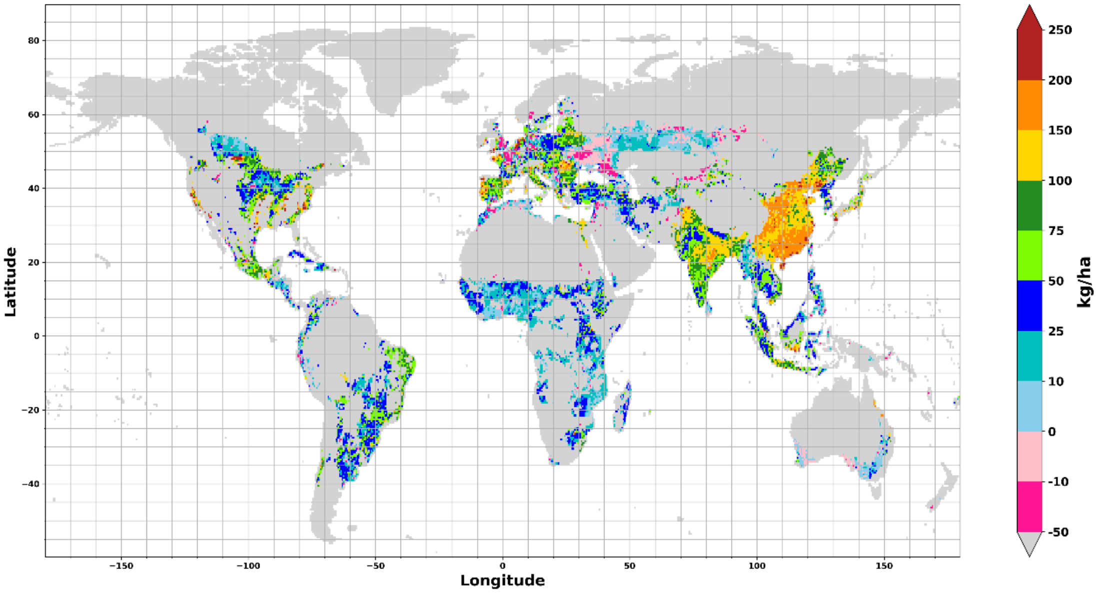

Figure S14. Soil Surface N budgets on SPAM cropland using IFA fertilizer with fraction on pastureland and volatilization losses subtracted.

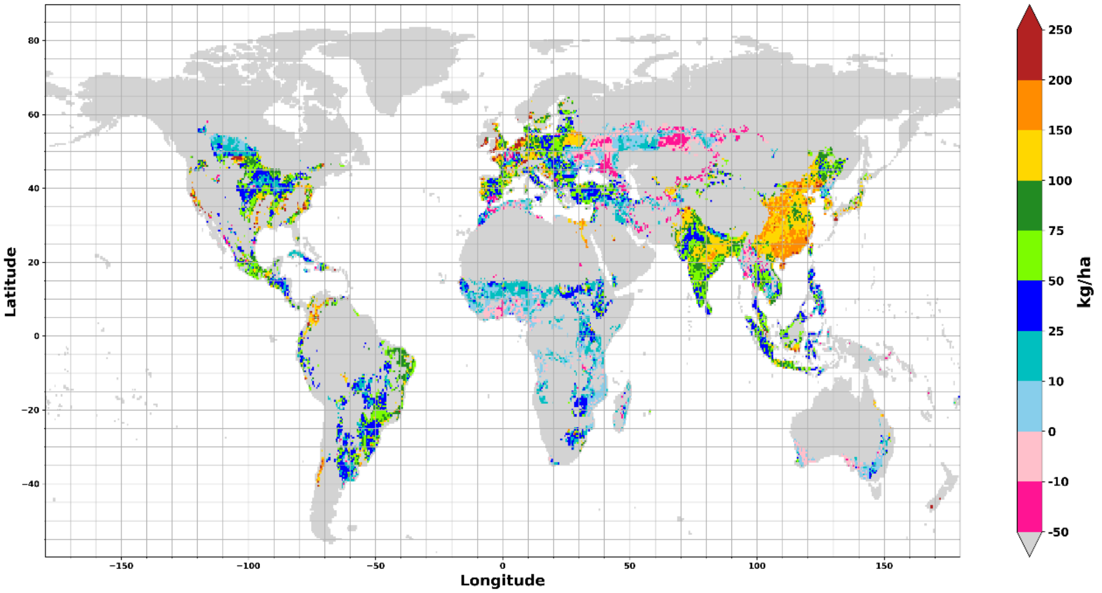

Figure S15. Soil Surface N budgets on SPAM cropland using FAO fertilizer with fraction on pastureland and volatilization losses subtracted.

Additional material used to derive at the results described in the paper, focusing on the role of different N inputs and outputs on the overall N indicator results are provided as Excel Tables in the data repository.

Data Set s02. Harvested Area, production, N inputs, N outputs and cropland area per region and crop category

Data Set s03. Harvested Area, production, N inputs, N outputs and cropland area per country and crop category

Data Set s04. Harvested Area, production, N inputs, N outputs and cropland area per country

Data Set s05. NUE comparison between Leip et al. (2009), Lassaletta et al. (2014), Bouwman et al. (2017) and variations of M3 and SPAM based calculations.
